# Pharmacogenomic biomarkers do not predict response to drotrecogin alfa in patients with severe sepsis

**DOI:** 10.1186/s13613-018-0353-2

**Published:** 2018-01-31

**Authors:** Djillali Annane, Jean-Paul Mira, Lorraine B. Ware, Anthony C. Gordon, Charles J. Hinds, David C. Christiani, Jonathan Sevransky, Kathleen Barnes, Timothy G. Buchman, Patrick J. Heagerty, Robert Balshaw, Nadia Lesnikova, Karen de Nobrega, Hugh F. Wellman, Mauricio Neira, Alexandra D. J. Mancini, Keith R. Walley, James A. Russell

**Affiliations:** 1 Service de Reanimation Medicale, Hopital R. Poincare, 104 Bd Raymond Poincare, 92380 Garches, France; 2 Sorbonne Paris Cité, Cochin Hotel-Dieu University Hospital Medical Intensive Care Unit, AP-HP, Université Paris Descartes, 75014 Paris, France; 3 Departments of Medicine and Pathology, Microbiology and Immunology, Vanderbilt University School of Medicine, 1161 21st Avenue South T1218 MCN, Nashville, TN 37232-2650 USA; 4 Section of Anaesthetics, Pain Medicine, and Intensive Care, Charing Cross Hospital, Imperial College London, Fulham Palace Road, London, W6 8RF UK; 5 Barts and The London School of Medicine, Queen Mary University of London, London, UK; 6 Harvard Medical School and School of Public Health, 665 Huntington Avenue, Building I Room 1401, Boston, MA 02115 USA; 7 Emory Center for Critical Care, Woodruff Health Sciences Center, Emory University, Atlanta, GA USA; 8 Division of Allergy and Clinical Immunology, Department of Medicine, Johns Hopkins University, Baltimore, MD USA; 9 Department of Biostatistics, University of Washington, F-600, Health Sciences Building, Office: H-665D HSB, Box 357232, Seattle, WA 98195-7232 USA; 10 Syreon Corporation, Vancouver, BC Canada; 11 Formerly with Sirius Genomics Inc, Vancouver, BC Canada; 12 Critical Care Research Laboratories, Centre for Heart Lung Innovation, St. Paul’s Hospital, University of British Columbia, Burrard Building, Rm 166 - 1081 Burrard St, Vancouver, BC V6Z 1Y6 Canada

**Keywords:** Drotrecogin alfa (activated), Activated protein C, Pharmacogenomics biomarker, Propensity score, Severe sepsis

## Abstract

**Purpose:**

To explore potential design for pharmacogenomics trials in sepsis, we investigate the interaction between pharmacogenomic biomarkers and response to drotrecogin alfa (activated) (DrotAA). This trial was designed to validate whether previously identified improved response polymorphisms (IRPs A and B) were associated with an improved response to DrotAA in severe sepsis.

**Methods:**

Patients with severe sepsis at high risk of death, who received DrotAA or not, with DNA available were included and matched to controls adjusting for age, APACHE II or SAPS II, organ dysfunction, ventilation, medical/surgical status, infection site, and propensity score (probability that a patient would have received DrotAA given their baseline characteristics). Independent genotyping and two-phase data transfer mitigated bias. The primary analysis compared the effect of DrotAA in IRP+ and IRP− groups on in-hospital 28-day mortality. Secondary endpoints included time to death in hospital; intensive care unit (ICU)-, hospital-, and ventilator-free days; and overall DrotAA treatment effect on mortality.

**Results:**

Six hundred and ninety-two patients treated with DrotAA were successfully matched to 1935 patients not treated with DrotAA. Genotyping was successful for 639 (DrotAA) and 1684 (nonDrotAA) matched patients. The primary hypothesis of a genotype-by-treatment interaction (assessed by conditional logistic regression analysis) was not significant (*P* = 0.30 IRP A; *P* = 0.78 IRP B), and there was no significant genotype by treatment interaction for any secondary endpoint.

**Conclusions:**

Neither IRP A nor IRP B predicted differential response to DrotAA on in-hospital 28-day mortality.

*ClinicalTrials.gov registration* NCT01486524

**Electronic supplementary material:**

The online version of this article (10.1186/s13613-018-0353-2) contains supplementary material, which is available to authorized users.

## Background

Pharmacogenomic biomarkers identify patients who have altered drug response according to their genotype. For example, use of pharmacogenomics of warfarin decreases risk of adverse events (severe hemorrhage) and increases efficacy [1–3]. Similarly, pharmacogenomics could be used to predict how patients with sepsis may respond to adjunctive therapies.

Treatment with DrotAA led to variable clinical responses in patients with sepsis. Then, one trial conducted in both severe sepsis and septic shock found a significant absolute risk reduction (ARR) of 6.1% in the 28-day mortality rate [4]. Other trials including only severe sepsis [5] or only septic shock [6, 7] failed to find survival benefit. One reason for the highly variable responses to DrotAA treatment observed in clinical trial may be related to genetic predisposition. In previous analyses, a combination of single nucleotide polymorphisms (SNPs) that defined two improve response polymorphisms (IRPs) was associated with a significant genotype-by-treatment interaction for effect of DrotAA on mortality.

Though DrotAA was withdrawn from the market by Ely Lilly, our overarching goal was to elaborate a pharmacogenomic approach using DrotAA as a practical example. Accordingly, the primary hypothesis was that IRP A and/or IRP B predict a differential DrotAA treatment effect in patients with severe sepsis and high risk of death.

## Methods

This is an abbreviated presentation of the methods of the current study because the details were published prior to undertaking analyses [8].

### Prior studies—background on selection of pharmacogenomic biomarkers for current study

To screen for genomic biomarkers, a Genome Wide Association Study (GWAS) of the PROWESS study [4] was performed (unpublished data) using DNA from 1446 patients to genotype approximately 1.2 million SNPs (Illumina^®^ Human1 M-Duo BeadChip). These results were taken forward to an independent cohort of patients who had septic shock, some of whom were treated with DrotAA and some of whom were not. This small replication cohort was drawn from St. Paul’s Hospital (SPH) and the Vasopressin and Septic Shock Trial (VASST) [9].

The replication cohort was used to confirm two IRPs. Two-SNP composite improved response polymorphisms (IRPs), A and B, were constructed. Patients were classified as IRP A+ or  − and IRP B+  or −  if they had one of both of the responsive genotype. For each IRP, individual patients were considered biomarker positive if they had the responsive genotype for either of the SNPs or for both of the SNPs in the IRP. The individual SNPs in each IRP were associated with a differential DrotAA treatment effect in PROWESS (derivation cohort) and replicated in the replication cohort (unpublished).

The two SNPs comprising IRP A were chosen based first on the alignment of direction and strength of their signals by analyzing the interaction of SNP and treatment effect on mortality in both the PROWESS study and the replication cohort. Secondly, these two SNPs were chosen based on biological plausibility linking the proteins coded by these genes to pathways of sepsis or pathways regarding mechanisms of action of DrotAA. The two SNPs of IRP A are RYR2 (ryanodine receptor 2 gene) rs684923 on chromosome 1 and ACIN1 (apoptotic chromatin condensation inducer 1 gene) rs3751501 on chromosome 14. The SNP of RYR2 could act to enhance efficacy of activated protein C on protection of endothelial permeability via its effects on endothelial protein C receptor and sphingosine-1-phosphate receptor 1 (S1P). When activated protein C (APC) binds to PAR1, this triggers more conversion of sphingosine to S1P, and this could decrease the amount of sphingosine and thus disinhibit the ryanodine receptor. We also suggest that this disinhibition of the ryanodine receptor by the actions of APC varies according to the genotype of the ryanodine receptor.

Phosphorylation of a residue (S422) inACIN1 (Acinus-S variant) by AKT (prosurvival kinase) completely inhibits cleavage of Acinus-S by caspase-3, abrogating the formation of fragment p17 which is essential for chromatin condensation during apoptosis. Apoptosis is increased in some tissues and cells (lymphocytes, dendritic cells, pulmonary and gut epithelial cells) and is decreased in other tissues and cells (neutrophils) in sepsis. This gene modulates apoptosis and activated protein C has anti-apoptotic actions apoptosis so we suggest that there could be an interaction between polymorphisms of ACIN1 and response to DrotAA. More specifically, the genetic variants rs3751501 (AA|AG), associated with increased ARR (absolute risk reduction) and coding for amino acid 478 F in ACIN1, would render ACIN1 constitutively nonphosphorylated at residue 478 F and hence constitutively nonphosphorylated at S422, leading to AKT-independent regulation of chromatin condensation by Acinus-S during apoptosis, because nonphosphorylated acinus-S would be constitutively cleavable by caspase-3.

The two SNPs comprising IRP B were chosen based solely on the strength of their signals in the PROWESS and replication cohorts. These two SNPs are SPATA7 (spermatogenesis associated 7 gene) rs3179969 on chromosome 14 and FLI1 (Friend leukemia virus integration 1 gene) rs640098 on chromosome 11.

For the replication cohort, the ARR was 19.7% for IRP A +  patients (95% confidence interval (CI) 2.2–37.1%), whereas for the IRP A  −  patients the ARR was −8.9% (95% CI −22.6–4.9%)(*p* = 0.018 unadjusted). The ARR was 21.2% for IRP B+  patients (95% CI 3.2–39.2%), whereas for the IRP B  −  patients the ARR was −5% (95% CI −18.2–8.2%)(*p* = 0.04 unadjusted).

### The current study—overall design

This was an international, multicenter, retrospective, controlled, outcome-blinded, genotype-blinded, and matched-patients study [8]. Retrospectively accessed DNA and clinical data were analyzed to validate the prespecified IRPs. Prospective aspects of this study were the genotyping of patients with regard to the IRPs and the statistical testing of the prespecified genotype hypothesis. Eight academic centers contributed the data and DNA from 10 cohorts (5 EU, 4 US, 1 Canada).

### Study population and treatment groups

Patients included in the current study (the INDICATED population) met prespecified eligibility criteria [8] and DrotAA-treated patients were matched to DrotAA-free patients. Eligibility criteria (aligned with the approved use of DrotAA in the USA and EU) were used to select the primary study population (INDICATED). Such patients with high risk of death represented common practice for use of DrotAA [10–14]. No patient in this study was part of a prospective randomized trial of DrotAA. Patients were treated according to standard care at their sites, and data and DNA samples collected at that time were retrospectively accessed for this study. All patients were enrolled after the Food and Drug Administration (FDA) or European Medicines Agency (EMA) approval of DrotAA.

### Matching DrotAA-treated to control patients

The current study incorporated a robust, well-accepted matching strategy. A propensity score of the estimated probability that a patient would have received DrotAA given their key baseline characteristics was calculated, and patients were selected as matches had to be within a prespecified tolerance on this score. Combining the use of propensity scores with covariate matching is superior to the use of either strategy alone [15]. The intended clinical variables for the calculation of the Mahalanobis distance and the reasons why these variables were chosen were described previously [8].

Syreon Corporation (a clinical research organization) conducted the study. A two-phase transfer of data from each center was performed to ensure that the selection of matched control patients was blinded and unbiased. Data transfer 1 included variables to confirm eligibility and to conduct the matching. Once the matching was achieved, the matched sets of treated and control patients were “locked” together. Then data transfer 2 (outcomes and genotypic data) was sent to Syreon.

### Genotyping

Genotyping for the IRP SNPs was done using a validated Taqman^®^-based analytical method and the laboratory was blinded to treatment and outcome. A 91-SNP ancestry informative marker (AIM) panel was genotyped by the GoldenGate^®^ analytical method.

### Statistical analysis

As previously noted [8], this study had 90% power to detect a treatment-by-IRP interaction assuming an absolute mortality reduction of 15% in the DrotAA-treated group compared to control in IRP + patients and with 1–2% difference in mortality between the treated and control groups in IRP- patients.

The primary analysis was done on the Matched-INDICATED population (comparing the effect of treatment in the IRP + and IRP- groups) by testing for the effect of the interaction between IRP and DrotAA treatment on the primary endpoint in a conditional logistic regression model, conditioning on the matching while incorporating the principal component scores from the 91-SNP AIM panel data (as covariates to control for potential population stratification). The primary endpoint was in-hospital mortality through Day 28 (i.e., patients were followed until hospital discharge or Day 28, whichever came first). Each of the primary analyses, one for IRP A and one for IRP B, was done as a two-sided test with *α* = 2.5% for an overall, Bonferroni-corrected, type I error rate of 5%.

## Results

**Fig. 1:**
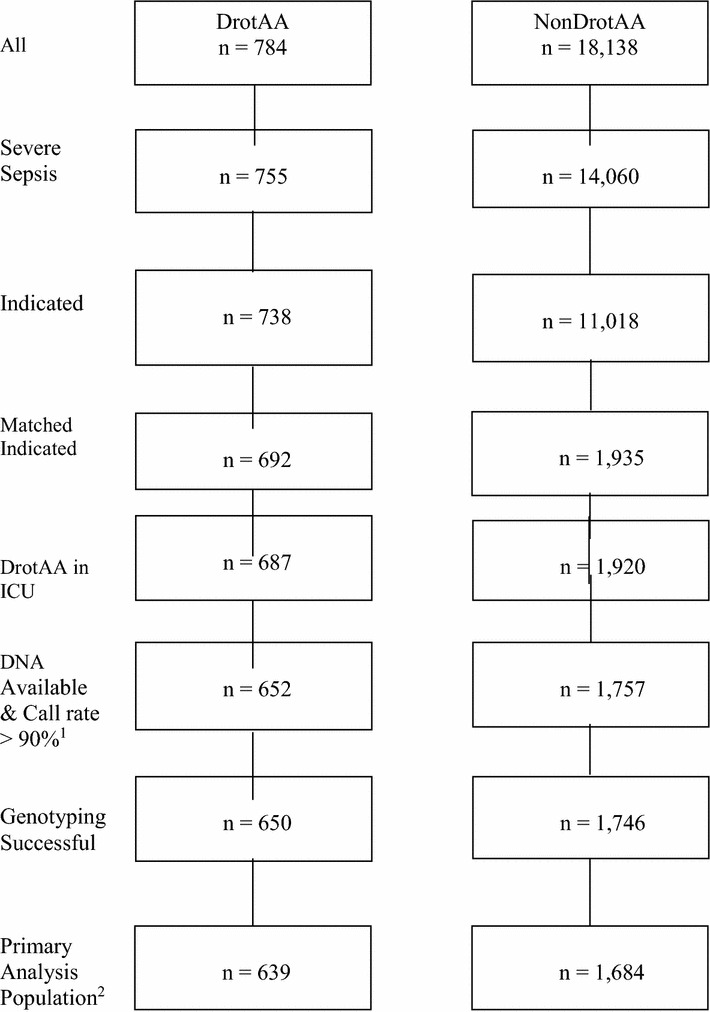
Patient enrollment in DrotAA and nonDrotAA groups. Superscript notes: ^1^Based on GoldenGate genotyping for AIM panel SNPs and research SNPs. ^2^Each matched set required 1 DrotAA-treated patient and 1–3 nonDrotAA-treated patients

Prior to matching patients treated with DrotAA differed significantly from nonDrotAA patients in many important baseline clinical respects (Additional file 1: Supplement Tables 1–6). After applying the eligibility criteria for the INDICATED population, there were 11,018 patients not treated with DrotAA from whom to choose matched controls for the 738 DrotAA-treated patients (ratio 15:1) (Fig. 1). Suitable matches were not available for 46 (6.2%) of the 738 INDICATED DrotAA-treated patients. Thus, after matching, the number of matched-INDICATED DrotAA-treated patients was 692. Overall the matched ratio was 2.8 control patients for every DrotAA-treated patient. Baseline clinical characteristics of the matched 692 DrotAA patients were similar to the 1,935 matched control patients **(**Table 1**).** The DrotAA group had higher proportion of coagulation dysfunction and a higher use of vasopressors (Table 1). Matching was done before genotype was known so it is relevant to observe that within the IRP A + and IRP A- and the IRP B+ and IRP B− genotype subgroups, DrotAA-treated patients were similar to matched control patients (Additional file 1: Supplement Tables 8–19). The Primary Analysis Population (PAP) included 639 DrotAA-treated patients and 1684 matched controls. The primary reasons why patients could not be included in the PAP were insufficient quantity of DNA or unsuccessful genotyping (Fig. 1, Additional file 1: Supplement Table 7).

**Table 1:** Baseline characteristics for ALL and matched-INDICATED populations by treatment

Baseline characteristics^a^	ALL patients		Matched-INDICATED	
	DrotAA (*n* = 784)	NonDrotAA (*n* = 18,138)	DrotAA (*n* = 692)	NonDrotAA (*n* = 1935)
Age (Mean)	58.4	60.4	59.0	59.1
SD	16.1	16.9	15.4	9.2
*P* value^b^	0.001		0.69	
Male (%)	59.7	59.9	60.1	61.2
*P* value	0.93		0.65	
APACHE II (mean)	26.1	21.1	25.8	25.5
SD	8.2	7.7	7.9	4.7
*P* value	< 0.0001		0.02	
SAPS II (mean)	58.1	47.8	59.1	58.5
SD	19.2	19.1	18.8	10.9
*P* value	< 0.0001		0.77	
*Origin of sepsis (%)*				
Nosocomial	8.4	4.7	7.2	12.3
Community acquired	38.6	14.3	39.7	34.7
Unknown	52.9	81.0	53.0	53.0
*P* value	0.003		< 0.0001	
*Anatomic site of primary infection (%)*				
Lung	51.5	23.4	52.9	46.8
Abdomen	12.2	6.9	13.2	12.7
CNS	1.3	0.6	0.9	0.7
Blood	3.4	2.0	3.0	3.5
Urinary tract	4.1	2.7	3.8	3.3
Unknown	22.1	60.2	21.1	29.0
Other	5.4	4.2	5.2	4.1
*P* value	0.0004		0.55	
*Use of vasopressors (%)*				
Yes	91.2	54.7	91.8	81.2
No	8.3	39.2	7.8	17.8
Unknown	0.5	6.1	0.4	1.0
*P* value	< 0.0001		< 0.0001	
*Mechanical ventilation (%)*				
Yes	76.3	72.3	75.7	76.4
No	6.4	19.5	6.2	5.2
Unknown	17.3	8.2	18.1	18.4
*P* value	< 0.0001		0.08	
*Number of organ systems with dysfunction (%)*				
0	1.4	9.9	0.0	0.0
1	2.2	23.8	0.1	0.8
2	20.7	30.8	21.0	22.0
3	33.0	19.4	35.1	34.0
4	24.9	11.3	26.3	29.4
5	13.6	4.1	13.9	11.7
6	4.2	0.7	3.6	2.2
*P* value	< 0.0001		0.82	
*Cardiovascular dysfunction (%)*				
Yes	96.0	66.9	98.7	98.3
No	2.4	30.2	1.0	1.0
Unknown	1.5	2.9	0.3	0.7
*P* value	< 0.0001		0.36	
*Pulmonary dysfunction (%)*				
Yes	92.6	69.5	95.4	95.3
No	5.1	18.8	3.9	3.4
Unknown	2.3	11.7	0.7	1.3
*P* value	< 0.0001		0.34	
*CNS dysfunction (%)*				
Yes	28.4	21.0	27.7	31.4
No	33.2	34.5	33.8	30.9
Unknown	38.4	44.5	38.4	37.7
*P* value	0.0003		0.01	
*Coagulation dysfunction (%)*				
Yes	31.4	14.0	31.8	25.0
No	65.4	68.7	66.3	72.6
Unknown	3.2	17.3	1.9	2.4
*P* value	< 0.0001		< 0.0001	
*Renal dysfunction (%)*				
Yes	63.3	29.4	65.6	61.7
No	33.9	54.1	32.4	36.6
Unknown	2.8	16.6	2.0	1.7
*P* value	< 0.0001		< 0.01	
*Hepatic dysfunction (%)*				
Yes	23.9	13.0	24.4	23.8
No	66.8	66.7	67.6	68.5
Unknown	9.3	20.3	7.9	7.7
*P* value	< 0.0001		0.71	

^a^Summary statistics for the NonDrotAA group were weighted to reflect the unequal numbers of DrotAA and NonDrotAA patients in each of the matched sets

^b^Descriptive *P* values are from clustered regression analysis using linear regression (numeric variables) or binary logistic regression (categorical variables) comparing the proportion of patients in the most frequent category between DrotAA vs NonDrotAA, clustering on the matched sets and with weights based on the number of patients in DrotAA and NonDrotAA matched sets. Patients in the unknown categories were excluded from the tests. No adjustments were made to account for multiple inference

### Effect of DrotAA on mortality

Irrespective of genotype, the ARR was in favor of DrotAA (estimated weighted mortality: DrotAA 25.1%, nonDrotAA 30.5%) (*P* = 0.006, Additional file 1: Supplement Table 21).

### Primary endpoint: genotype-by-DrotAA treatment interaction

The primary hypothesis of a genotype by DrotAA treatment interaction assessed by conditional logistic regression analysis for IRP A was not significant (*P* = 0.30, Table 2), and the direction of the effect was opposite to what had been expected as shown by the negative parameter estimate for the interaction term (Additional file 1: Supplement Tables 20–21). The IRP B result was also not significant (*P* = 0.78, Table 2), and the direction of effect was opposite to what had been expected. There was a therapeutic benefit of DrotAA treatment in IRP A- negative and IRP B- negative patients.

**Table 2:** Primary efficacy analysis—conditional logistic regression model including AIM Panel PCs

Factor/effect	Estimate	SE	Odds ratio (OR) estimate	OR 95% CI		*P* value^c^
				Lower	Upper	
*Primary efficacy analysis for IRP A*						
IRP A*Treatment Interaction^a^	− 0.31	0.303	0.731	0.403	1.324	0.30
IRP A+: DrotAA versus NonDrotAA	0.11	0.245	1.112	0.688	1.799	0.66
IRP A−: DrotAA versus NonDrotAA	0.42	0.145	1.522	1.145	2.024	< 0.01
DrotAA: IRP A+ versus IRP A−	− 0.30	0.258	0.740	0.446	1.228	0.24
NonDrotAA: IRP A+ versus IRP A−	0.01	0.150	1.013	0.755	1.359	0.93
AIM Panel PCs^b^						0.14
*Primary efficacy Analysis for IRP B*						
IRP B*Treatment Interaction^a^	− 0.09	0.325	0.912	0.482	1.722	0.78
IRP B+: DrotAA versus NonDrotAA	0.23	0.277	1.257	0.730	2.162	0.41
IRP B−: DrotAA versus NonDrotAA	0.32	0.138	1.379	1.052	1.807	0.02
DrotAA: IRP B+ versus IRP B−	− 0.19	0.285	0.829	0.475	1.449	0.51
NonDrotAA: IRP B+ versus IRP B−	− 0.09	0.156	0.910	0.670	1.236	0.55
AIM Panel PCs^b^						0.14

Analysis for IRP A involved 376 discordant matched sets from a total of 637 matched sets

Analysis for IRP B involved 372 discordant matched sets from a total of 634 matched sets

^a^The interaction odds ratio is a ratio of odds ratios

^b^A total of 10 AIM Panel PCs were included which accounted for 33.9% of the variance in the AIM Panel data for the Matched-INDICATED Primary Analysis Population based on all cohorts

^c^*P* values from conditional logistic regression partial likelihood ratio tests for the IRP*Treatment Interaction and the combined AIM Panel PCs; all other *P* values are from Wald Chi-square tests

Neither IRP A nor IRP B predicted ICU and hospital survival rates or the destination at hospital discharge. At hospital discharge, the mortality rates were 32.2 and 36.2% for DrotAA group and nonDrotAA groups, respectively. Approximately 19% of patients were discharged home, 13% went to long-term care facilities, 9% went to another acute care hospital, and discharge location was unknown for the remainder (24–27%).

### Sensitivity analyses with common matching variables included as covariates

The inclusion of the common matching variables as covariates in the conditional logistic regression model did not change any of the conclusions regarding IRP A, IRP B, or the overall DrotAA effect (Table 3). We used conditional logistic regression and included common matching variables (age, APACHE II score or SAPS II score, and respiratory dysfunction (yes/no)) as covariates to correct for residual imbalances across IRP genotype subgroups. Neither self-reported race nor genetically determined continent of origin impacted the overall DrotAA treatment effect or the IRP*Treatment interactions in conditional logistic regression models.

**Table 3:** Secondary analysis with common matching variables included as covariates—conditional logistic regression for differential treatment effects of IRP A and IRP B on mortality

Factor/effect	Estimate	SE	Odds Ratio (OR) estimate	OR 95% CI		*P* value^c^
				Lower	Upper	
*Analysis for IRP A*						
IRP A*Treatment Interaction^a^	−0.34	0.304	0.710	0.391	1.289	0.26
IRP A+ : DrotAA versus NonDrotAA	0.08	0.246	1.087	0.671	1.761	0.74
IRP A−: DrotAA versus NonDrotAA	0.43	0.146	1.532	1.151	2.039	< 0.01
DrotAA: IRP A+ versus IRP A−	−0.32	0.259	0.725	0.436	1.205	0.22
NonDrotAA: IRP A+ versus IRP A−	0.02	0.150	1.022	0.761	1.372	0.89
AIM Panel PCs^b^						0.13
Common covariates used in matching						0.74
Age (per year)	−0.02	0.024	0.976	0.930	1.023	0.30
APACHE II (per point)^d^	0.05	0.080	1.054	0.901	1.233	0.51
SAPS II (per point)^d^	0.01	0.041	1.006	0.929	1.091	0.88
Respiratory dysfunction (Yes vs. No)	0.86	0.778	2.371	0.515	10.90	0.27
*Analysis for IRP B*						
IRP B*Treatment Interaction^a^	−0.10	0.325	0.908	0.480	1.719	0.77
IRP B+: DrotAA versus NonDrotAA	0.22	0.278	1.251	0.726	2.155	0.42
IRP B−: DrotAA versus NonDrotAA	0.32	0.138	1.377	1.050	1.806	0.02
DrotAA: IRP B+ vs IRP B−	−0.19	0.285	0.826	0.472	1.443	0.50
NonDrotAA: IRP B+ vs IRP B−	−0.10	0.157	0.909	0.668	1.236	0.54
AIM panel PCs^b^						0.15
Common covariates used in matching						0.75
Age (per year)	−0.02	0.024	0.976	0.931	1.024	0.32
APACHE II (per point)^d^	0.04	0.080	1.045	0.894	1.223	0.58
SAPS II (per point)^d^	−0.00	0.041	0.997	0.919	1.081	0.94
Respiratory dysfunction (Yes vs. No)	0.12	0.935	1.127	0.180	7.050	0.90

Analysis for IRP A involved 376 discordant matched sets from a total of 637 matched sets

Analysis for IRP B involved 372 discordant matched sets from a total of 634 matched sets

^a^The interaction odds ratio is a ratio of odds ratios

^b^A total of 10 AIM Panel PCs were included which account for 33.9% of the variance in the AIM Panel data for the Matched-INDICATED Primary Analysis Population based on all cohorts

^c^*P* values from conditional logistic regression partial likelihood ratio tests for the IRP*Treatment Interaction and the combined AIM Panel PCs; all other *P* values are from Wald Chi-square tests

^d^Sites with APACHE II scores were analyzed with 0’s for SAPS II scores, and vice versa

### Sensitivity analyses according to high APACHE II/SAPS II scores

As in PROWESS [4] the overall effect of DrotAA differed according to severity of illness (Table 4). Greater severity of illness was defined by APACHE II ≥25 and by SAPS II ≥54. ARR based on crude mortality rates was 10.3% in the high severity of illness and—1.8% in the low severity of illness subgroups.

**Table 4:** Secondary analyses for effects of high APACHE II/SAPS II scores on differential treatment effects of IRP A and IRP B on mortality—conditional logistic regression models including AIM panel PCs

Factor/effect	Estimate	SE	Odds Ratio (OR) estimate	OR 95% CI		*P* value^c^
				Lower	Upper	
*Analysis for IRP A*						
Treatment	−0.15	0.258	0.861	0.519	1.427	0.56
IRP A	−0.08	0.278	0.926	0.537	1.597	0.78
IRP A by treatment interaction^a^	−0.01	0.535	0.989	0.347	2.819	0.98
High APACHE II/SAPS II Scores	−0.06	0.352	0.940	0.472	1.872	0.86
High APACHE II/SAPS II scores by treatment interaction^a^	0.82	0.315	2.273	1.226	4.215	< 0.01
High APACHE II/SAPS II scores by IRP A Interaction^a^	0.15	0.329	1.164	0.611	2.218	0.65
IRP A by treatment by high APACHE II/SAPS II Scores	−0.42	0.651	0.659	0.184	2.360	0.52
AIM Panel PCs^b^						< 0.01
*Analysis for IRP B*						
Treatment	−0.13	0.251	0.880	0.538	1.438	0.61
IRP B	0.14	0.280	1.153	0.666	1.999	0.61
IRP B by treatment interaction^a^	−0.09	0.541	0.912	0.316	2.635	0.87
High APACHE II/SAPS II Scores	0.10	0.344	1.100	0.561	2.157	0.78
High APACHE II/SAPS II Scores by Treatment^a^	0.63	0.302	1.869	1.033	3.379	0.04
High APACHE II/SAPS II Scores by IRP B Interaction^a^	−0.35	0.338	0.704	0.363	1.366	0.30
IRP B by treatment by high APACHE II/SAPS II Scores	0.09	0.677	1.092	0.290	4.118	0.90
AIM Panel PCs^b^						< 0.01

Analysis for IRP A involves 376 discordant matched sets from a total of 637 matched sets. Analysis for IRP B involves 372 discordant matched sets from a total of 634 matched sets

^a^The interaction odds ratio is a ratio of odds ratios

^b^A total of 10 AIM Panel PCs have been included which account for 33.9% of the variance in the AIM Panel data for the Matched-INDICATED Primary Analysis Population based on all cohorts

^c^*P* values are from Wald Chi-square tests; the test of the combined AIM Panel PCs is the sum of the individual Wald Chi-square tests

### Sensitivity analyses of individual IRP A and IRP B SNPs

None of the IRP A nor IRP B SNPs was significantly associated with treatment effect when analyzed individually.

### Secondary endpoints

**Fig. 2:**
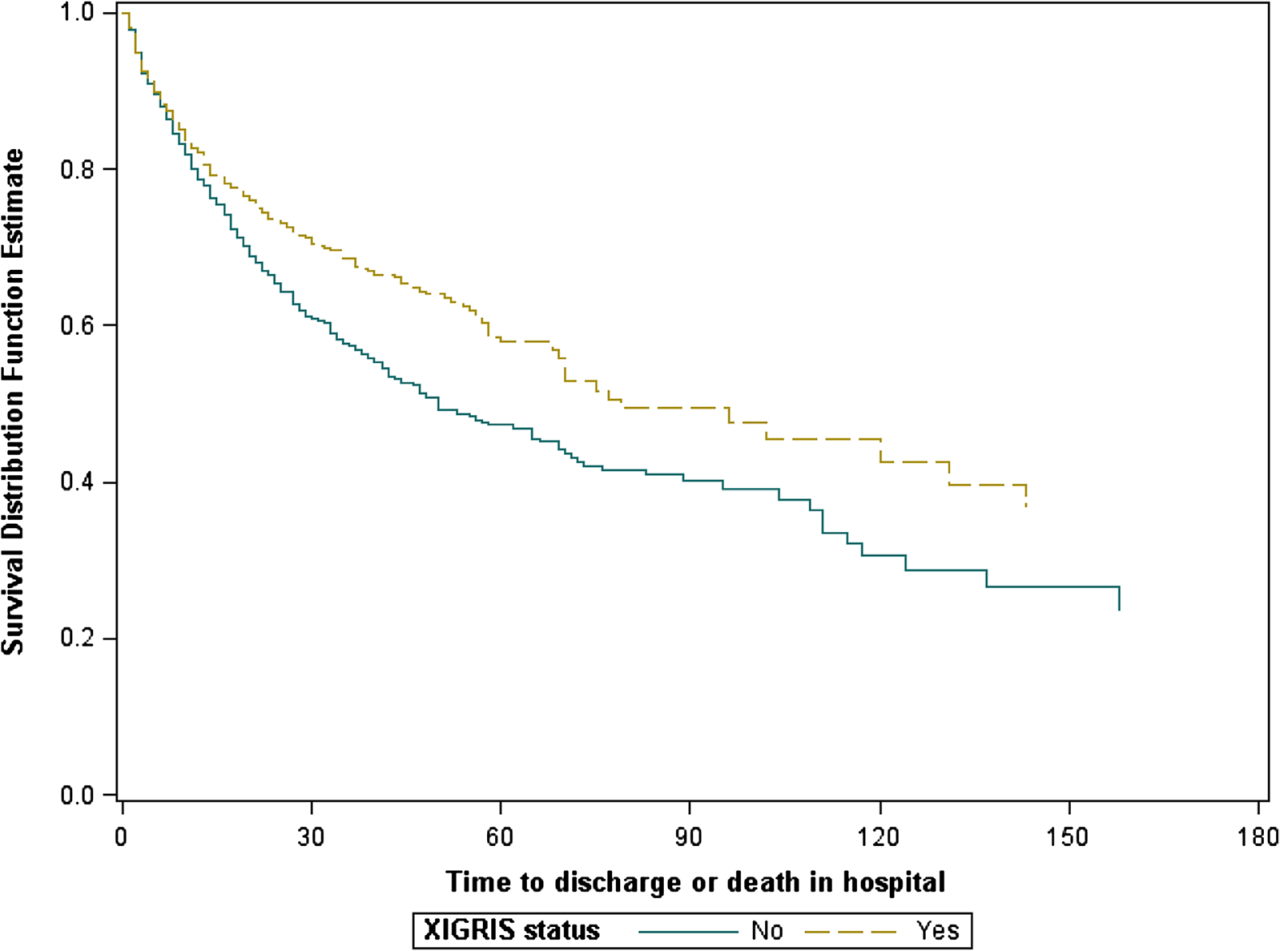
Kaplan–Meier curves showing estimated 28-day mortality rates of approximately 28% for the DrotAA (Xigris) group and 38% for the nonDrotAA group

Time to death in hospital analyses showed a beneficial DrotAA treatment effect but no additional benefit to knowing IRP A or IRP B genotype (Fig. 2). None of the secondary efficacy endpoints analyses (time to death in hospital, ICU-free days, hospital-free days, and mechanical-ventilator-free days) showed a significant genotype-by-treatment interaction (Additional file 1: Supplement Tables 22–26).

## Discussion

This international, multicenter, retrospective, nonrandomized, controlled, outcome-blinded, genotype-blinded, and matched-patients study found that neither IRP A nor IRP B predicted differential DrotAA treatment effects on in-hospital mortality through Day 28 in patients with severe sepsis. Furthermore, there was no significant genotype by treatment interaction for any of the secondary endpoints. Nonetheless, as yet undiscovered, other genotypes might accurately predict response to DrotAA.

Despite the negative results of the genotype by DrotAA treatment interaction, we suggest that this study provided a potential design for future evaluations of pharmacogenomic biomarkers of drugs and devices for use in sepsis. Most recent and ongoing sepsis trials include DNA biobanking. We propose that these trials systematically explore potential experimental treatment interaction with genomic biomarkers. If any positive interaction, a confirmatory RCT with biomarker-based stratification of randomization would then be conducted.

The possible explanations for the negative findings are that the prior discovery studies were false positive results that did not validate because the biology assumptions were incorrect for the biology SNPs and that the statistics were misleading for the statistically chosen SNPs. The lessons to be learned for future biomarker trials include need for greater validation in prior studies before the pivotal trials are done, perhaps focus only on statistically chosen SNPs, and access to prior trials in which SNPs were assessed (such as PROWESS in the current example).

We believe that this is the largest study of predictive genomic biomarkers for any drug used in sepsis. DrotAA treatment was associated with similar survival benefit in the IRP + and IRP- genotype subgroups. Self-reported ethnicity, genetically assigned continent of origin, and high APACHE II/SAPS II analyses did not show predictive genetic IRP effects in any subgroup. Exploratory analyses of the four individual IRP SNPs also did not show any predictive biomarker effects. Similarly, neither IRP A nor IRP B was predictive of differential DrotAA treatment effects on secondary efficacy endpoints. Only the IRP B + genotype was associated with significantly longer duration in hospital in DrotAA-treated patients compared with the IRP B- genotype.

Strengths of this study were first, identification of a well-defined and clinically appropriate patient population (on-label as defined by the approved indications for DrotAA in both the EU and the USA). Secondly, we minimized patient selection bias by using matching to select appropriate patients for the control group to overcome the lack of randomization to DrotAA treatment. DrotAA was typically given to younger patients with greater severity of disease in clinical practice. Therefore, simply comparing the mortality rates in all DrotAA-treated patients to all nonDrotAA-treated patients in the 10 cohorts would have been invalid. The considerations in designing this study were to use either design-based or analysis-based methods to control for characteristics that differed at baseline between treatment groups. We chose to use a design-based approach of matching to tightly control for key measures, so that the control group was comparable to the treated group. This also permitted efficient use of resources since only a subsample of all possible controls would be needed for detailed genotypic evaluation. The low use of DrotAA in clinical practice for patients with severe sepsis in the included cohorts permitted this matched-patients study to be conducted. If the drug had been used most of the time in the eligible patients, then it would have been very difficult to match appropriate nonDrotAA-treated patients as matched controls. The matching process was successful in achieving well-balanced study groups, and balance between the two study groups was achieved across IRP genotype subgroups (IRP+, IRP−), thus minimizing confounding of any biomarker effects (IRP*treatment interaction) by baseline differences.

The third strength of the study was quality of data, genotyping, and ancestry-control. Extensively reviewed phenotypic data from all 10 cohorts were combined in a common database. Genotyping of the IRP SNPs was conducted using a validated Taqman-based method. A panel of ancestry informative markers (AIM panel with 91 SNPs) was genotyped using a qualified GoldenGate genotyping method. Quality criteria were applied to the genomic data based on per-sample and per-SNP call rates and Hardy–Weinberg equilibrium testing. These strengths are confirmed by finding similar results to PROWESS in that we were successful in overcoming the patient selection bias that can occur in a retrospective, nonrandomized study.

DrotAA has been suggested to be beneficial in the most severe patients, and especially in those with advanced coagulopathy. Coagulation dysfunction was diagnosed in only 31.8% of the treated patients, which might partly explain the negative results. However, we clarify that we chose our inclusion criteria to align with the drug label and indicated use of DrotAA. Our overall hypothesis was that a genomic marker(s) would identify responders to DrotAA and that a diagnostic kit could then be developed to help clinicians decide whom to treat with DrotAA.

The SNP selection and interaction test results of the prior studies could have been influenced by the so-called winner’s curse. If adequate methods had been taken to deal with the winner’s curse, then the sample size requirement for adequate power may have increased because we would not have over-estimated the assumed effect size of the IRP.

Although the present results are negative, it is useful to discuss the feasibility of IRP detection (including time and cost) and treatment allocation based on the presence of such IRPs. The genotype can be measured now in 40–60 min (Cepheid for example), even in the Emergency or ICU setting, and location(s) a time frame reasonable for IRP detection in sepsis and septic shock. The cost would depend upon the technology platform cost, the reagent costs, and the clinical value of the test results based on studies of cost–benefit of the test versus not having the test.

Limitations of our study were that the treatment assignment of DrotAA was not blinded nor randomized, outcomes were obtained retrospectively from databases, and we could not assess safety due to lack of adequate data. Examples of some confounders that we could not control for are physician and judgement regarding patient prognosis and eligibility for treatment with DrotAA, individual ICU or hospital policies regarding use of DrotAA, and other underlying disease that we did not capture. We did not specifically select patients that were randomized in DrotAA trials because we did not have access to the trials of DrotAA that collected DNA such as PROWESS [4, 16]. Sepsis is a complex trait, so it is likely that future research regarding better patient selection for treatment will consider several phenotypic biomarkers (not just focusing on genetic diversity) with or without genotype assessments.

Why were our IRP A and IRP B genotypes not predictive? It is possible that our method of selection of SNPs for IRP A and IRP B was inadequate. We used an underpowered replication cohort to refine SNPs of interest from the PROWESS study and we incorporated both SNPs based on pure statistical signal in the replication cohort as well as SNPs that were chosen based on both strength of signal in the replication cohort as well as biological plausibility. Our study highlights the importance and difficulty of validation of predictive genotype-base biomarkers in severe sepsis.

Our finding of a beneficial treatment effect of DrotAA in the current study differs from PROWESS SHOCK [6] and from the APROCCHS trial [7] (in which there was no treatment effect) and may reflect selection bias. Additionally, our study was not randomized, included an earlier era of patients, and we found higher mortality rates than in PROWESS SHOCK.

## Conclusions

Neither IRP A nor IRP B predicted a differential DrotAA treatment effect in patients with severe sepsis and high risk of death.

## Additional file

**Additional file 1.** Additional supplementary tables and figures.

## Abbreviations

PROWESS: prospective recombinant human activated protein C worldwide evaluation in severe sepsis
IRP: improved response polymorphism
DROTAA: drotrecogin alfa (activated)
APACHE II: acute physiology and chronic health evaluation
SAPS: simplified acute physiology score
SNP: single nucleotide polymorphism
ICU: intensive care unit
ARR: absolute risk reduction
GWAS: Genome Wide Association Study
SPH: St. Paul’s Hospital
VASST: vasopressin and septic shock trial
RYR2: ryanodine receptor 2 gene
ACIN1: apoptotic chromatin condensation inducer 1 gene
S1P: sphingosine-1-phosphate receptor 1
APC: activated protein C
PAR1: protease-activated receptor 1
AKT: prosurvival kinase
SPATA7: spermatogenesis associated 7 gene
FLI1: friend leukemia virus integration 1 gene
FDA: food and drug administration
EMA: European medicines agency
AIM: ancestry informative marker
PAP: primary analysis population
APROCCHS: activated protein C and corticosteroids for human septic shock
