# Supplementary material for: Pharmacogenomic biomarkers do not predict response to drotrecogin alfa in patients with severe sepsis
Source: Ann Intensive Care. 2018 Jan 31;8:16. doi: 10.1186/s13613-018-0353-2 (PMC5792380; doi:10.1186/s13613-018-0353-2)
Supplement: Supplementary file 1 — Additional file 1. Additional supplementary tables and figures. [file 13613_2018_353_MOESM1_ESM.doc]

**PHARMACOGENOMIC BIOMARKERS DO NOT PREDICT RESPONSE TO DROTRECOGIN ALFA IN PATIENTS WITH SEVERE SEPSIS**

Djillali Annane, MD, Jean-Paul Mira, MD, Lorraine B. Ware, MD, Anthony C. Gordon, MD, Charles Hinds, MD, David C. Christiani, MD, Jonathan Sevransky, MD, Kathleen Barnes, PhD, Timothy G. Buchman, MD, Patrick J. Heagerty, PhD, Robert Balshaw, PhD, Nadia Lesnikova, MSc, Karen de Nobrega, Hugh F. Wellman, MSc, Mauricio Neira, PhD, Alexandra D.J. Mancini, MSc, Keith R. Walley, MD, James A. Russell, MD

**SUPPLEMENT**

**METHODS**

A second study population with severe sepsis (non-INDICATED) was selected in which severe sepsis patients did not necessarily meet the high risk of death criteria (Table 2 of Annane et al[^1^](#_ENREF_1)). The INDICATED and non-INDICATED populations were merged to form the severe sepsis (SEVSEP) population.

DrotAA-treated patients were given DrotAA in accordance with local regulatory approvals and clinical practice.

**Matching**

We controlled for differences in standard of care among centers and over time by matching DrotAA-treated patients to controls (DrotAA-free patients) enrolled within two years in the same cohort. After meeting eligibility criteria, control patients were matched with DrotAA-treated patients using a computerized optimal matching algorithm incorporating baseline demographic and disease characteristics that had been identified a priori as likely influencing first, the decision to prescribe DrotAA or second, the probability of death. The number of matched control patients for each DrotAA-treated patient varied from one to three to increase the precision in the estimation of the differences between groups. The matching strategy combined minimum-distance matching using “calipers” that forced the matches for selected variables to fall within specified tolerances. Individual variables were used to compute a multivariate distance (Mahalanobis distance).

The propensity score was estimated using a logistic regression model for treatment group using the matching variables included in the calculation of Mahalanobis distances across all centers, plus a categorical variable for center. We tested for interaction between age and APACHE II[^2^](#_ENREF_2) or SAPS II[^3^](#_ENREF_3) scores and for interactions between age and each of the four organ dysfunctions (cardiovascular, respiratory, hematologic, and renal). If individual interactions were significant at the 0.05 level then these interaction terms were included in the propensity score model.

Calipers were applied to selected key variables to ensure close matches. For age, a maximum 5-year difference was chosen. For APACHE II scores, we expected scores to be mainly 20-40 and so we required a 2 point caliper that yields a difference in predicted mortality of about 7% at the low end and 1% at the high end. For SAPS II, a 4-point caliper was applied (comparable with the 2-point APACHE II caliper[^2^](#_ENREF_2).

The propensity score caliper was set at 0.6 standard deviations (of the average propensity score) because this often decreases bias[^4^](#_ENREF_4) .

Almost all DrotAA-treated patients had shock and thus instead of including cardiovascular dysfunction as a distance variable, it became a caliper requiring an exact match.

**Missing Data**

No imputation of missing data was done to satisfy eligibility criteria. To support selection of matched patients (once deemed eligible), missing data for up to two matching variables were imputed for an individual patient. Missing data was imputed using available data from that same center if for any matching variable, the proportion of missing values per center was <30% for the INDICATED population; if higher, imputation was not to be done. No missing data imputations were allowed for age, APACHE II or SAPS II scores.

**Genotyping**

The plate layout of DNA samples was randomized to avoid systematic bias. DrotAA-treated patients and their matched controls were assigned to the same plate to ensure tight control of external factors within each set of matched patients.

A panel of 91 Ancestry Informative Marker (AIM) SNPs was genotyped using the Illumina GoldenGate® analytical method. This method for ancestry assignments (using the STRUCTURE software package) adequately identifies patients of European, African and Asian ancestry.

**Statistical Analysis**

The target sample size was >700 DrotAA-treated patients in the INDICATED population. If 750 DrotAA-treated patients and approximately 1500 matched control patients were enrolled, the current study would have adequate power when testing two hypotheses, IRP A and IRP B, corrected for multiplicity testing.

Estimates of the effect of treatment within each IRP status subgroup were presented as odds ratios and the 95% CIs from the conditional logistic regression analysis. ARRs for each IRP status subgroup and their 95% CIs based on weighted mortality estimates are shown for descriptive purposes. Secondary analyses included matching variables as covariates in the regression model to adjust for residual imbalances and possible confounding. Additionally, an ethnicity subgroup analysis was used to investigate the three-way interaction among ethnicity, treatment, and IRP in a matched conditional logistic regression model.

For secondary endpoints, stratified Cox regression was used to estimate time to death in hospital (censored at discharge). Conditional logistic regression was used to estimate the log-odds of mortality as a function of IRP, treatment group, interaction between IRP and treatment, conditioned on the matched sets. Mechanical ventilator-free days, ICU-free days, and hospital-free days (all through Day 28) were analyzed using Poisson regression models.

**RESULTS**

**AIM Panel**

We controlled for possible population stratification (ethnicity effects) by evaluating 10 AIM principal component (PC) scores that were included in the regression model; these 10 AIM PCs accounted for 33.9% of the variance in the AIM panel data for the Matched-INDICATED population.

**Crude Mortality Rates**

The crude mortality rates for the DrotAA and nonDrotAA treatment group by IRP A and IRP B genotypes (Table 20) do not consider the matched sets or adjust for the variable number of nonDrotAA patients in the matched sets. Therefore it is not the best method for summarizing the treatment effects based on the matched-patients study design. Nevertheless, it illustrates the numbers of patients who died within subgroups. The DrotAA treatment ARRs were very similar within IRP A (IRP A+ 5.1, IRP A- 4.9) and IRP B subgroups (IRP B+ 7.5, IRP B- 3.7). Thus, DrotAA treatment decreased mortality in all genotype subgroups; the IRPs did not predict differential treatment effects.

**Weighted Mortality Rates**

Weighted mortality is based on a clustered binomial regression model, which does address the matched sets study design and the variable weighting for the matched nonDrotAA patients in the matched sets. Therefore this prespecified approach is more appropriate for summarizing the treatment effects based on the matched-patients study design. This secondary analysis method was to provide mortality estimates and ARRs, which are not provided from the primary analysis method (conditional logistic regression). The DrotAA treatment ARRs were very similar within the IRP A subgroups (IRP A+ 5.1, IRP A- 5.7) and within the IRP B subgroups (IRP B+ 7.7, IRP B- 4.2) (Table 21).

**Exploratory Analyses of Individual IRP A and IRP B SNPs**

Having shown that neither of the two-SNP IRPs (A and B) was predictive, unplanned exploratory analyses were performed to understand whether any of the four individual IRP SNPs predicted differential DrotAA treatment effects. Three types of analyses were performed for each of the four SNPs – the primary analysis (conditional logistic regression), crude mortality, and secondary analysis (clustered binomial regression for estimates of weighted mortality rates and ARRs). These three analyses were also done using stratification on high APACHE II/SAPS II scores. The IRP-by-Treatment interactions were not significant for any of the four individual IRP SNPs. Therefore none of these SNPs is a predictive biomarker for differential DrotAA treatment effects**.** Inclusion of the high APACHE II/SAPS II term for the four individual IRP A and B SNPs did not change these conclusions.

**ICU-Free, Hospital-Free and Mechanical Ventilator-Free Days through Day 28**

The clustered Poisson regression analyses for the secondary efficacy endpoint of ICU-free days through Day 28 (Table 22) showed no significant IRP-by-Treatment interactions. There was a statistically significant effect of DrotAA treatment on ICU-free days; the DrotAA group had fewer ICU-free days (mean 7.8, median 2) compared to the nonDrotAA group (mean 10.5, median 10, *P*<0.0001) (Tables 25-26). Similarly, there was no significant IRP-by-Treatment interaction with hospital-free days (Table 23). DrotAA was associated with significantly fewer hospital-free days (mean 3.7, median 0) compared to the nonDrotAA group (mean 5.0, median 0, *P*<0.0001) (Tables 25-26). Neither IRP A nor IRP B was a predictive biomarker for differential DrotAA treatment effect on ventilator-free days (Table 24). A statistically significant effect of DrotAA treatment was identified with this analysis (*P*=0.0222). The DrotAA group had fewer mechanical ventilator-free days (mean 11.2, median 10) compared to the nonDrotAA group (mean 12.4, median 13, *P*=0.0198) (Tables 25-26).

1. Demographics for ALL, INDICATED, and Matched-INDICATED Populations by Treatment

|  | | | | | | |
| --- | --- | --- | --- | --- | --- | --- |
|  | ALL Patients | | INDICATED | | Matched-INDICATED | |
| Demographic Characteristics^a^ | DrotAA (n=784) | NonDrotAA (n=18138) | DrotAA (n=738) | NonDrotAA (n=11018) | DrotAA (n=692) | NonDrotAA (n=1935) |
|  |  |  |  |  |  |  |
| Age |  |  |  |  |  |  |
| Mean | 58.42 | 60.44 | 58.59 | 61.89 | 59.01 | 59.07 |
| SD | 16.059 | 16.940 | 15.860 | 16.299 | 15.396 | 9.157 |
| Min | 16.0 | 6.0 | 20.0 | 18.0 | 20.0 | 18.0 |
| Q1 | 48.0 | 50.0 | 48.0 | 51.0 | 48.0 | 49.0 |
| Median | 59.0 | 62.0 | 59.0 | 64.0 | 60.0 | 60.0 |
| Q3 | 71.0 | 74.0 | 71.0 | 75.0 | 71.0 | 71.0 |
| Max | 107.0 | 101.0 | 107.0 | 98.0 | 98.0 | 96.0 |
| *P*-value^b^ | 0.0011 |  | <.0001 |  | 0.6908 |  |
|  |  |  |  |  |  |  |
| Gender (%) |  |  |  |  |  |  |
| Male | 59.7 | 59.9 | 59.2 | 60.5 | 60.1 | 61.2 |
| Female | 40.2 | 40.1 | 40.7 | 39.5 | 39.7 | 38.8 |
| Unknown | 0.1 | 0.0 | 0.1 | 0.0 | 0.1 | 0.1 |
| *P*-value^b^ | 0.9271 |  | 0.5268 |  | 0.6547 |  |
|  |  |  |  |  |  |  |
| Recorded Race (%) |  |  |  |  |  |  |
| American Indian or Alaska Native | 0.5 | 1.0 | 0.5 | 0.9 | 0.6 | 0.5 |
| Asian | 1.4 | 1.1 | 1.5 | 1.1 | 1.6 | 1.5 |
|  |  |  |  |  |  |  |
| Black or African American | 6.8 | 6.5 | 6.9 | 6.8 | 6.9 | 6.9 |
| Native Hawaiian or Other Pacific Islander | 0.0 | 0.0 | 0.0 | 0.0 | 0.0 | 0.0 |
| White | 76.0 | 83.9 | 76.4 | 80.9 | 77.6 | 77.0 |
| Unknown | 15.2 | 7.4 | 14.5 | 10.1 | 13.2 | 13.8 |
| Other | 0.1 | 0.1 | 0.1 | 0.2 | 0.1 | 0.2 |
| *P*-value^b^ | 0.4068 |  | 0.5964 |  | 0.9942 |  |
|  |  |  |  |  |  |  |
| Recorded Ethnicity (%) |  |  |  |  |  |  |
| Hispanic or Latino | 2.0 | 2.5 | 2.0 | 2.4 | 1.7 | 1.8 |
| Not Hispanic or Latino | 82.8 | 90.0 | 83.5 | 87.4 | 85.1 | 84.2 |
| Unknown | 15.2 | 7.5 | 14.5 | 10.2 | 13.2 | 14.0 |
| *P*-value^b^ | 0.6335 |  | 0.6076 |  | 0.9181 |  |
|  |  |  |  |  |  |  |

^a^Percentages have been calculated relative to the number of patients in the population. In the matched population, summary statistics for the NonDrotAA group have been weighted to reflect the unequal numbers of DrotAA and NonDrotAA patients in each of the matched sets.

^b^Descriptive *P*-values for the unmatched populations are from T-tests comparing the means (numeric variables) or Pearson Chi-square tests (categorical variables) comparing the proportion of patients in the most frequent category between DrotAA vs. NonDrotAA. For the matched population, *P*-values are from clustered regression analysis using linear regression (numeric variables) or binary logistic regression (categorical variables) comparing the proportion of patients in the most frequent category between DrotAA vs NonDrotAA, clustering on the matched sets and with weights based on the number of patients in DrotAA and NonDrotAA matched sets. Patients in the unknown categories have been excluded from the tests. No adjustments have been made to account for multiple inference.

1. Baseline Medical History or Comorbidities for ALL, INDICATED, and Matched-INDICATED Populations by Treatment

| Percentage of Patients | | | | | | |
| --- | --- | --- | --- | --- | --- | --- |
|  | ALL Patients | | INDICATED | | Matched-INDICATED | |
| Preexisting Condition or Comorbidity^a^ | DrotAA (n=784) | NonDrotAA (n=18138) | DrotAA (n=738) | NonDrotAA (n=11018) | DrotAA (n=692) | NonDrotAA (n=1935) |
|  |  |  |  |  |  |  |
| Hypertension |  |  |  |  |  |  |
| Yes | 15.8 | 22.6 | 16.0 | 21.1 | 16.2 | 15.0 |
| No | 17.3 | 17.9 | 16.4 | 14.8 | 16.0 | 18.0 |
| Unknown | 66.8 | 59.5 | 67.6 | 64.1 | 67.8 | 67.0 |
| *P*-value^b^ | 0.0102 |  | 0.0042 |  | 0.1944 |  |
|  |  |  |  |  |  |  |
| Myocardial infarction |  |  |  |  |  |  |
| Yes | 4.2 | 9.3 | 4.2 | 8.7 | 3.3 | 4.7 |
| No | 44.0 | 32.4 | 44.6 | 31.9 | 45.5 | 45.1 |
| Unknown | 51.8 | 58.3 | 51.2 | 59.3 | 51.2 | 50.3 |
| *P*-value^b^ | <.0001 |  | <.0001 |  | 0.1881 |  |
|  |  |  |  |  |  |  |
| Congestive heart failure |  |  |  |  |  |  |
| Yes | 6.5 | 9.7 | 6.5 | 10.9 | 6.2 | 11.1 |
| No | 64.5 | 37.7 | 64.6 | 38.9 | 64.7 | 62.5 |
| Unknown | 29.0 | 52.6 | 28.9 | 50.1 | 29.0 | 26.4 |
| *P*-value^b^ | <.0001 |  | <.0001 |  | 0.0003 |  |
|  |  |  |  |  |  |  |
| COPD |  |  |  |  |  |  |
| Yes | 8.3 | 8.0 | 8.7 | 7.7 | 8.7 | 10.7 |
| No | 61.7 | 43.8 | 61.7 | 42.4 | 61.8 | 60.7 |
| Unknown | 30.0 | 48.2 | 29.7 | 49.9 | 29.5 | 28.6 |
| *P*-value^b^ | 0.0223 |  | 0.0667 |  | 0.0967 |  |
|  |  |  |  |  |  |  |
| Diabetes |  |  |  |  |  |  |
| Yes | 20.9 | 19.9 | 20.6 | 22.1 | 20.8 | 20.6 |
| No | 59.2 | 39.7 | 60.2 | 45.4 | 60.3 | 60.6 |
| Unknown | 19.9 | 40.4 | 19.2 | 32.5 | 18.9 | 18.8 |
| *P*-value^b^ | 0.0002 |  | 0.0003 |  | 0.9086 |  |
|  |  |  |  |  |  |  |
| Pancreatitis |  |  |  |  |  |  |
| Yes | 1.1 | 0.7 | 1.1 | 0.7 | 1.0 | 1.1 |
| No | 20.7 | 15.0 | 20.6 | 13.8 | 21.2 | 21.7 |
| Unknown | 78.2 | 84.4 | 78.3 | 85.6 | 77.7 | 77.2 |
| *P*-value^b^ | 0.5498 |  | 0.8118 |  | 0.8259 |  |
|  |  |  |  |  |  |  |
| Renal dialysis |  |  |  |  |  |  |
| Yes | 2.4 | 2.5 | 2.6 | 3.4 | 2.6 | 4.0 |
| No | 42.3 | 37.0 | 43.1 | 39.7 | 44.4 | 43.8 |
| Unknown | 55.2 | 60.6 | 54.3 | 56.8 | 53.0 | 52.2 |
| *P*-value^b^ | 0.4949 |  | 0.1266 |  | 0.0749 |  |
|  |  |  |  |  |  |  |
| Cirrhosis |  |  |  |  |  |  |
| Yes | 2.8 | 3.1 | 2.7 | 3.5 | 2.9 | 5.5 |
| No | 65.4 | 43.5 | 66.0 | 49.5 | 66.2 | 64.4 |
| Unknown | 31.8 | 53.4 | 31.3 | 47.0 | 30.9 | 30.1 |
| *P*-value^b^ | 0.0212 |  | 0.0179 |  | 0.0113 |  |
|  |  |  |  |  |  |  |
| Other liver disease |  |  |  |  |  |  |
| Yes | 6.0 | 4.3 | 6.2 | 5.0 | 5.8 | 7.4 |
| No | 21.6 | 25.5 | 21.0 | 30.7 | 21.4 | 20.9 |
| Unknown | 72.4 | 70.2 | 72.8 | 64.3 | 72.8 | 71.7 |
| *P*-value^b^ | 0.0025 |  | 0.0006 |  | 0.0077 |  |
|  |  |  |  |  |  |  |
| Malignancy |  |  |  |  |  |  |
| Yes | 14.7 | 12.6 | 14.5 | 12.8 | 14.6 | 16.7 |
| No | 64.7 | 60.4 | 65.2 | 60.6 | 65.5 | 64.2 |
| Unknown | 20.7 | 27.1 | 20.3 | 26.6 | 19.9 | 19.1 |
| *P*-value^b^ | 0.4243 |  | 0.6442 |  | 0.2289 |  |
|  |  |  |  |  |  |  |
| Immunocompromised |  |  |  |  |  |  |
| Yes | 16.5 | 9.8 | 16.5 | 11.7 | 14.6 | 16.7 |
| No | 55.2 | 54.9 | 55.7 | 52.6 | 65.5 | 64.2 |
| Unknown | 28.3 | 35.3 | 27.8 | 35.7 | 19.9 | 19.1 |
| *P*-value^b^ | <.0001 |  | 0.0070 |  | 0.2289 |  |
|  |  |  |  |  |  |  |

^a^Percentages have been calculated relative to the number of patients in the population. In the matched population, summary statistics for the NonDrotAA group have been weighted to reflect the unequal numbers of DrotAA and NonDrotAA patients in each of the matched sets.

^b^Descriptive *P*-values for the unmatched populations are from Pearson Chi-square tests comparing the proportion of patients in the most frequent category between DrotAA vs. NonDrotAA. For the matched population, *P*-values are from clustered binary logistic regression comparing the proportion of patients in the most frequent category between DrotAA vs NonDrotAA, clustering on the matched sets and with weights based on the number of patients in DrotAA and NonDrotAA matched sets. Patients in the unknown categories have been excluded from the tests. No adjustments have been made to account for multiple inference.

1. Baseline Infection Characteristics for ALL, INDICATED, and Matched-INDICATED Populations by Treatment

| Percentage of Patients | | | | | | |
| --- | --- | --- | --- | --- | --- | --- |
|  | ALL Patients | | INDICATED | | Matched-INDICATED | |
| Baseline Infection Parameters^a^ | DrotAA (n=784) | NonDrotAA (n=18138) | DrotAA (n=738) | NonDrotAA (n=11018) | DrotAA (n=692) | NonDrotAA (n=1935) |
|  |  |  |  |  |  |  |
| Proven or Suspected Infection |  |  |  |  |  |  |
| Proven | 75.8 | 46.7 | 77.1 | 58.4 | 76.3 | 66.5 |
| Suspected | 17.2 | 27.7 | 16.9 | 27.9 | 17.3 | 26.2 |
| Unknown | 7.0 | 25.6 | 6.0 | 13.7 | 6.4 | 7.3 |
| *P*-value^b^ | <.0001 |  | <.0001 |  | <.0001 |  |
|  |  |  |  |  |  |  |
| Proven Infection^c^ | n=594 | n=8468 | n=569 | n=6439 | n=528 | n=1287 |
| Gram- | 29.0 | 24.9 | 29.5 | 27.6 | 29.2 | 32.0 |
| *P*-value^b^ | 0.0274 |  | 0.3131 |  | 0.2136 |  |
| Gram+ | 39.4 | 31.8 | 39.9 | 34.2 | 39.4 | 36.4 |
| *P*-value^b^ | 0.0001 |  | 0.0063 |  | 0.1545 |  |
| Gram Variable | 0.3 | 0.1 | 0.4 | 0.1 | 0.4 | 0.1 |
| *P*-value^b^ | 0.0856 |  | 0.0803 |  | 0.1723 |  |
| Fungi | 2.5 | 4.1 | 2.5 | 4.5 | 2.5 | 5.7 |
| *P*-value^b^ | 0.0540 |  | 0.0226 |  | 0.0026 |  |
| Virus | 2.5 | 1.7 | 2.6 | 1.6 | 2.8 | 2.0 |
| *P*-value^b^ | 0.1192 |  | 0.0823 |  | 0.2432 |  |
| Unknown | 9.6 | 19.3 | 10.0 | 18.6 | 10.2 | 14.5 |
| *P*-value^b^ | <.0001 |  | <.0001 |  | 0.0025 |  |
| Other | 0.0 | 0.4 | 0.0 | 0.5 |  | 0.8 |
| *P*-value^b^ | 0.1218 |  | 0.0972 |  |  |  |
|  |  |  |  |  |  |  |
| Suspected Infection | n=135 | n=5022 | n=125 | n=3069 | n=120 | n=507 |
| Culture requested but No IV Antibiotics given | 9.6 | 14.5 | 10.4 | 14.6 | 10.8 | 8.3 |
| IV Antibiotics given but No Culture requested | 71.1 | 71.6 | 69.6 | 68.9 | 70.8 | 70.5 |
| Both Culture requested and IV Antibiotics given | 19.3 | 13.8 | 20.0 | 16.5 | 18.3 | 21.2 |
| *P*-value^b^ | 0.0731 |  | 0.2963 |  | 0.4294 |  |
|  |  |  |  |  |  |  |
| Origin of Sepsis |  |  |  |  |  |  |
| Nosocomial | 8.4 | 4.7 | 7.5 | 6.2 | 7.2 | 12.3 |
| Community acquired | 38.6 | 14.3 | 40.1 | 19.4 | 39.7 | 34.7 |
| Unknown | 52.9 | 81.0 | 52.4 | 74.4 | 53.0 | 53.0 |
| *P*-value^b^ | 0.0028 |  | 0.0004 |  | <.0001 |  |
|  |  |  |  |  |  |  |
| Anatomic Site of Primary Infection |  |  |  |  |  |  |
| Lung | 51.5 | 23.4 | 52.7 | 31.2 | 52.9 | 46.8 |
| Abdomen | 12.2 | 6.9 | 12.6 | 9.4 | 13.2 | 12.7 |
| CNS | 1.3 | 0.6 | 1.2 | 0.5 | 0.9 | 0.7 |
| Blood | 3.4 | 2.0 | 3.3 | 2.7 | 3.0 | 3.5 |
| Urinary tract | 4.1 | 2.7 | 4.1 | 3.4 | 3.8 | 3.3 |
| Unknown | 22.1 | 60.2 | 20.9 | 47.5 | 21.1 | 29.0 |
| Other | 5.4 | 4.2 | 5.3 | 5.2 | 5.2 | 4.1 |
| *P*-value^b^ | 0.0004 |  | 0.0008 |  | 0.5539 |  |
|  |  |  |  |  |  |  |

^a^Percentages have been calculated relative to the number of patients in the population, with the exception of Proven Infection types (Gram +/-, etc) and Suspected Infection evidence (culture requested, etc), where the numbers of patients with proven or suspected infections have been used, respectively. In the matched population, summary statistics for the NonDrotAA group have been weighted to reflect the unequal numbers of DrotAA and NonDrotAA patients in each of the matched sets.

^b^Descriptive *P*-values for the unmatched populations are from Pearson Chi-square tests comparing the proportion of patients in the most frequent category between DrotAA vs. NonDrotAA. For the matched population, *P*-values are from clustered binary logistic regression comparing the proportion of patients in the most frequent category between DrotAA vs NonDrotAA, clustering on the matched sets and with weights based on the number of patients in DrotAA and NonDrotAA matched sets. Patients in the unknown categories have been excluded from the tests. No adjustments have been made to account for multiple inference.

^c^Not mutually exclusive categories.

1. Baseline Organ Dysfunctions for ALL, INDICATED, and Matched-INDICATED Populations by Treatment

|  | | | | | | |
| --- | --- | --- | --- | --- | --- | --- |
|  | ALL Patients | | INDICATED | | Matched-INDICATED | |
| Baseline Organ Dysfunction Parameter^a^ | DrotAA (n=784) | NonDrotAA (n=18138) | DrotAA (n=738) | NonDrotAA (n=11018) | DrotAA (n=692) | NonDrotAA (n=1935) |
|  |  |  |  |  |  |  |
| Number of Organ Systems with Dysfunction (%) |  |  |  |  |  |  |
| 0 | 1.4 | 9.9 | 0.0 | 0.0 | 0.0 | 0.0 |
| 1 | 2.2 | 23.8 | 0.3 | 4.7 | 0.1 | 0.8 |
| 2 | 20.7 | 30.8 | 21.1 | 43.9 | 21.0 | 22.0 |
| 3 | 33.0 | 19.4 | 34.3 | 28.4 | 35.1 | 34.0 |
| 4 | 24.9 | 11.3 | 26.0 | 16.3 | 26.3 | 29.4 |
| 5 | 13.6 | 4.1 | 14.0 | 5.8 | 13.9 | 11.7 |
| 6 | 4.2 | 0.7 | 4.3 | 0.9 | 3.6 | 2.2 |
| *P*-value^b^ | <.0001 |  | <.0001 |  | 0.8201 |  |
|  |  |  |  |  |  |  |
| Number of Organ Systems with Dysfunction |  |  |  |  |  |  |
| Mean | 3.36 | 2.14 | 3.45 | 2.77 | 3.44 | 3.36 |
| SD | 1.212 | 1.323 | 1.108 | 1.028 | 1.081 | 0.623 |
| Min | 0.0 | 0.0 | 1.0 | 0.0 | 1.0 | 1.0 |
| Q1 | 3.0 | 1.0 | 3.0 | 2.0 | 3.0 | 3.0 |
| Median | 3.0 | 2.0 | 3.0 | 3.0 | 3.0 | 3.0 |
| Q3 | 4.0 | 3.0 | 4.0 | 3.0 | 4.0 | 4.0 |
| Max | 6.0 | 6.0 | 6.0 | 6.0 | 6.0 | 6.0 |
| *P*-value^b^ | <.0001 |  | <.0001 |  | 0.0126 |  |
|  |  |  |  |  |  |  |
| Cardiovascular (%) |  |  |  |  |  |  |
| Yes | 96.0 | 66.9 | 98.6 | 88.1 | 98.7 | 98.3 |
| No | 2.4 | 30.2 | 1.1 | 10.8 | 1.0 | 1.0 |
| Unknown | 1.5 | 2.9 | 0.3 | 1.1 | 0.3 | 0.7 |
| *P*-value^b^ | <.0001 |  | <.0001 |  | 0.3608 |  |
|  |  |  |  |  |  |  |
| Pulmonary (%) |  |  |  |  |  |  |
| Yes | 92.6 | 69.5 | 95.0 | 85.1 | 95.4 | 95.3 |
| No | 5.1 | 18.8 | 4.1 | 10.4 | 3.9 | 3.4 |
| Unknown | 2.3 | 11.7 | 0.9 | 4.5 | 0.7 | 1.3 |
| *P*-value^b^ | <.0001 |  | <.0001 |  | 0.3401 |  |
|  |  |  |  |  |  |  |
| CNS (%) |  |  |  |  |  |  |
| Yes | 28.4 | 21.0 | 28.9 | 25.5 | 27.7 | 31.4 |
| No | 33.2 | 34.5 | 32.9 | 30.1 | 33.8 | 30.9 |
| Unknown | 38.4 | 44.5 | 38.2 | 44.4 | 38.4 | 37.7 |
| *P*-value^b^ | 0.0003 |  | 0.7486 |  | 0.0124 |  |
|  |  |  |  |  |  |  |
| Coagulation (%) |  |  |  |  |  |  |
| Yes | 31.4 | 14.0 | 32.2 | 18.3 | 31.8 | 25.0 |
| No | 65.4 | 68.7 | 65.7 | 71.6 | 66.3 | 72.6 |
| Unknown | 3.2 | 17.3 | 2.0 | 10.1 | 1.9 | 2.4 |
| *P*-value^b^ | <.0001 |  | <.0001 |  | <.0001 |  |
|  |  |  |  |  |  |  |
| Renal (%) |  |  |  |  |  |  |
| Yes | 63.3 | 29.4 | 65.9 | 41.9 | 65.6 | 61.7 |
| No | 33.9 | 54.1 | 32.0 | 48.4 | 32.4 | 36.6 |
| Unknown | 2.8 | 16.6 | 2.2 | 9.7 | 2.0 | 1.7 |
| *P*-value^b^ | <.0001 |  | <.0001 |  | 0.0033 |  |
|  |  |  |  |  |  |  |
| Hepatic (%) |  |  |  |  |  |  |
| Yes | 23.9 | 13.0 | 24.7 | 18.5 | 24.4 | 23.8 |
| No | 66.8 | 66.7 | 67.2 | 67.4 | 67.6 | 68.5 |
| Unknown | 9.3 | 20.3 | 8.1 | 14.2 | 7.9 | 7.7 |
| *P*-value^b^ | <.0001 |  | 0.0012 |  | 0.7091 |  |
|  |  |  |  |  |  |  |

^a^Percentages have been calculated relative to the number of patients in the population. In the matched population, summary statistics for the NonDrotAA group have been weighted to reflect the unequal numbers of DrotAA and NonDrotAA patients in each of the matched sets.

^b^Descriptive *P*-values for the unmatched populations are from T-tests comparing the means (numeric variables) or Pearson Chi-square tests (categorical variables) comparing the proportion of patients in the most frequent category between DrotAA vs. NonDrotAA. For the matched population, *P*-values are from clustered regression analysis using linear regression (numeric variables) or binary logistic regression (categorical variables) comparing the proportion of patients in the most frequent category between DrotAA vs NonDrotAA, clustering on the matched sets and with weights based on the number of patients in DrotAA and NonDrotAA matched sets. For Number of Organ Systems with Dysfunction, the distribution was split at the modal count into higher versus lower counts. Patients in the unknown categories have been excluded from the tests. No adjustments have been made to account for multiple inference.

1. Baseline Risk of Death Characterization for ALL, INDICATED, and Matched-INDICATED Populations by Treatment

|  | ALL Patients | | INDICATED | | Matched-INDICATED | |
| --- | --- | --- | --- | --- | --- | --- |
| Baseline Risk of Death Parameter^a^ | DrotAA (n=784) | NonDrotAA (n=18138) | DrotAA (n=738) | NonDrotAA (n=11018) | DrotAA (n=692) | NonDrotAA (n=1935) |
|  |  |  |  |  |  |  |
| APACHE II |  |  |  |  |  |  |
| N | 515 | 11077 | 493 | 7176 | 478 | 1339 |
| Mean | 26.05 | 21.05 | 26.16 | 23.07 | 25.79 | 25.51 |
| SD | 8.212 | 7.676 | 8.080 | 7.236 | 7.888 | 4.748 |
| Min | 8.0 | 0.0 | 8.0 | 1.0 | 8.0 | 1.0 |
| Q1 | 20.0 | 16.0 | 20.0 | 18.0 | 20.0 | 20.0 |
| Median | 25.0 | 21.0 | 25.0 | 23.0 | 25.0 | 25.0 |
| Q3 | 32.0 | 26.0 | 32.0 | 27.5 | 31.0 | 31.0 |
| Max | 47.0 | 52.0 | 47.0 | 52.0 | 47.0 | 48.0 |
| *P*-value^b^ | <.0001 |  | <.0001 |  | 0.0245 |  |
|  |  |  |  |  |  |  |
| SAPS II |  |  |  |  |  |  |
| N | 480 | 10658 | 449 | 5683 | 424 | 1186 |
| Mean | 58.08 | 47.81 | 59.13 | 54.06 | 59.08 | 58.50 |
| SD | 19.192 | 19.094 | 19.051 | 17.941 | 18.811 | 10.892 |
| Min | 17.0 | 0.0 | 17.0 | 8.0 | 20.0 | 18.0 |
| Q1 | 43.0 | 34.0 | 44.0 | 41.0 | 44.0 | 44.0 |
| Median | 57.0 | 46.0 | 58.0 | 53.0 | 58.0 | 57.0 |
| Q3 | 71.0 | 60.0 | 72.0 | 65.0 | 72.0 | 69.0 |
| Max | 111.0 | 138.0 | 111.0 | 138.0 | 111.0 | 111.0 |
| *P*-value^b^ | <.0001 |  | <.0001 |  | 0.7678 |  |
|  |  |  |  |  |  |  |
| High Risk of Death Criteria Met (%) |  |  |  |  |  |  |
| APACHE II or SAPS II only |  |  |  |  | 0.3 | 0.9 |
| MOD only |  |  |  |  | 40.3 | 41.6 |
| Both |  |  |  |  | 59.4 | 57.4 |
| *P*-value^b^ |  |  |  |  | 0.1394 |  |
|  |  |  |  |  |  |  |

^a^Percentages have been calculated relative to the number of patients in the population. In the matched population, summary statistics for the NonDrotAA group have been weighted to reflect the unequal numbers of DrotAA and NonDrotAA patients in each of the matched sets.

^b^Descriptive *P*-values for the unmatched populations are from T-tests comparing the means (numeric variables) or Pearson Chi-square tests (categorical variables) comparing the proportion of patients in the most frequent category between DrotAA vs. NonDrotAA. For the matched population, *P*-values are from clustered regression analysis using linear regression (numeric variables) or binary logistic regression (categorical variables) comparing the proportion of patients in the most frequent category between DrotAA vs NonDrotAA, clustering on the matched sets and with weights based on the number of patients in DrotAA and NonDrotAA matched sets. Patients in the unknown categories have been excluded from the tests. No adjustments have been made to account for multiple inference.

MOD = multiple organ dysfunction

1. Use of Vasopressors and Mechanical Ventilation in ALL, INDICATED, and Matched-INDICATED Populations by Treatment

Percentage of Patients

|  | ALL Patients | | INDICATED | | Matched-INDICATED | |
| --- | --- | --- | --- | --- | --- | --- |
| Supportive Treatment^a^ | DrotAA (n=784) | NonDrotAA (n=18138) | DrotAA (n=738) | NonDrotAA (n=11018) | DrotAA (n=692) | NonDrotAA (n=1935) |
|  |  |  |  |  |  |  |
| Use of Vasopressors |  |  |  |  |  |  |
| Yes | 91.2 | 54.7 | 92.1 | 70.4 | 91.8 | 81.2 |
| No | 8.3 | 39.2 | 7.5 | 27.8 | 7.8 | 17.8 |
| Unknown | 0.5 | 6.1 | 0.4 | 1.8 | 0.4 | 1.0 |
| *P*-value^b^ | <.0001 |  | <.0001 |  | <.0001 |  |
|  |  |  |  |  |  |  |
| Mechanical Ventilation |  |  |  |  |  |  |
| Yes | 76.3 | 72.3 | 75.7 | 76.1 | 75.7 | 76.4 |
| No | 6.4 | 19.5 | 6.1 | 13.6 | 6.2 | 5.2 |
| Unknown | 17.3 | 8.2 | 18.2 | 10.3 | 18.1 | 18.4 |
| *P*-value^b^ | <.0001 |  | <.0001 |  | 0.0798 |  |
|  |  |  |  |  |  |  |

^a^Percentages have been calculated relative to the number of patients in the population. In the matched population, summary statistics for the NonDrotAA group have been weighted to reflect the unequal numbers of DrotAA and NonDrotAA patients in each of the matched sets.

^b^Descriptive *P*-values for the unmatched populations are from Pearson Chi-square tests comparing the proportion of patients in the most frequent category between DrotAA vs. NonDrotAA. For the matched population, *P*-values are from clustered binary logistic regression comparing the proportion of patients in the most frequent category between DrotAA vs NonDrotAA, clustering on the matched sets and with weights based on the number of patients in DrotAA and NonDrotAA matched sets. Patients in the unknown categories have been excluded from the tests. No adjustments have been made to account for multiple inference.

1. Disposition of Patients after Matching

| **Reason for Removal from Matched-INDICATED Analysis Population^a^** |  | DrotAA |  | NonDrotAA |
| --- | --- | --- | --- | --- |
|  |  |  |  |  |
| **Matched-INDICATED Population** |  | **692** |  | **1935** |
|  |  |  |  |  |
| DrotAA given in different ICU admission, matched set dropped |  | 5 |  | 15 |
|  |  |  |  |  |
| DrotAA-treated patient had no DNA, matched set dropped |  | 16 |  | 44 |
|  |  |  |  |  |
| NonDrotAA-treated patient had no DNA |  | - |  | 27 |
|  |  |  |  |  |
| Patient DNA Call Rate <90%^b^ |  | 19 |  | 92 |
|  |  |  |  |  |
| Genotyping unsuccessful for both IRP A and IRP B |  | 2 |  | 11 |
|  |  |  |  |  |
| Primary endpoint (in-hospital mortality through Day 28) missing |  | 4 |  | 7 |
|  |  |  |  |  |
| Incomplete matched set^c^ |  | 7 |  | 55 |
|  |  |  |  |  |
| Subtotal of patients removed from Primary Analysis Population |  | 53 |  | 251 |
|  |  |  |  |  |
| **Primary Analysis Population**  **(for either IRP A or B)** |  | **639** |  | **1684** |
|  |  |  |  |  |

^a^Reasons are mutually exclusive categories, listed hierarchically

^b^Based on GoldenGate genotyping for AIM panel SNPs and research SNPs

^c^Each matched set required 1 DrotAA-treated patient and 1-3 nonDrotAA-treated patients

1. Demographics for Matched-INDICATED Primary Analysis Population by IRP A Genotype and Treatment

|  | | | | | | |
| --- | --- | --- | --- | --- | --- | --- |
|  | IRP A+ | | IRP A- | | All Genotypes Combined | |
| Demographic Characteristics^a^ | DrotAA (n=197) | NonDrotAA (n=517) | DrotAA (n=440) | NonDrotAA (n=1159) | DrotAA (n=639) | NonDrotAA (n=1684) |
|  |  |  |  |  |  |  |
| Age |  |  |  |  |  |  |
| Mean | 59.61 | 59.25 | 59.31 | 59.55 | 59.41 | 59.45 |
| SD | 14.822 | 9.361 | 15.342 | 9.278 | 15.190 | 9.305 |
| Min | 23.0 | 18.0 | 20.0 | 18.0 | 20.0 | 18.0 |
| Q1 | 50.0 | 49.0 | 49.0 | 50.0 | 49.0 | 50.0 |
| Median | 60.0 | 60.0 | 60.5 | 60.0 | 60.0 | 60.0 |
| Q3 | 71.0 | 72.0 | 71.0 | 71.0 | 71.0 | 72.0 |
| Max | 88.0 | 90.0 | 98.0 | 96.0 | 98.0 | 96.0 |
| *P*-value^b^ | 0.5713 |  | 0.3453 |  | 0.9441 |  |
|  |  |  |  |  |  |  |
| Gender (%) |  |  |  |  |  |  |
| Male | 62.4 | 59.3 | 58.9 | 61.3 | 59.9 | 60.6 |
| Female | 37.6 | 40.6 | 41.1 | 38.6 | 40.1 | 39.3 |
| Unknown | 0.0 | 0.2 | 0.0 | 0.1 | 0.0 | 0.1 |
| *P*-value^b^ | 0.4059 |  | 0.3848 |  | 0.7401 |  |
|  |  |  |  |  |  |  |
| Recorded Race (%) |  |  |  |  |  |  |
| American Indian or Alaska Native | 0.5 | 0.2 | 0.5 | 0.5 | 0.5 | 0.4 |
| Asian | 3.0 | 3.2 | 1.1 | 0.9 | 1.7 | 1.6 |
| Black or African American | 7.6 | 8.5 | 6.6 | 5.7 | 6.9 | 6.7 |
| White | 72.6 | 74.2 | 79.8 | 78.5 | 77.6 | 77.2 |
| Unknown | 16.2 | 13.7 | 11.8 | 14.0 | 13.1 | 13.8 |
| Other | 0.0 | 0.2 | 0.2 | 0.4 | 0.2 | 0.3 |
| *P*-value^b^ | 0.8225 |  | 0.6441 |  | 0.8900 |  |
|  |  |  |  |  |  |  |
| Recorded Ethnicity (%) |  |  |  |  |  |  |
| Hispanic or Latino | 2.0 | 2.6 | 1.8 | 1.3 | 1.9 | 1.7 |
| Not Hispanic or Latino | 81.7 | 83.5 | 86.4 | 84.5 | 85.0 | 84.3 |
| Unknown | 16.2 | 13.9 | 11.8 | 14.2 | 13.1 | 14.0 |
| *P*-value^b^ | 0.6979 |  | 0.4660 |  | 0.7809 |  |
|  |  |  |  |  |  |  |
| Genetically Determined Continent of Origin (%) |  |  |  |  |  |  |
| European | 76.6 | 78.5 | 84.3 | 84.4 | 81.7 | 82.4 |
| African | 6.1 | 6.2 | 4.8 | 4.5 | 5.2 | 5.0 |
| Asian | 6.6 | 5.1 | 2.3 | 1.9 | 3.6 | 2.9 |
| Other | 10.7 | 10.1 | 8.6 | 9.1 | 9.5 | 9.7 |
| *P*-value^b^ | 0.5447 |  | 0.9534 |  | 0.6606 |  |
|  |  |  |  |  |  |  |

^a^Percentages have been calculated relative to the number of patients in the population. Summary statistics for the NonDrotAA group have been weighted to reflect the unequal numbers of DrotAA and NonDrotAA patients in each of the matched sets.

^b^Descriptive *P*-values are from clustered regression analysis using linear regression (numeric variables) or binary logistic regression (categorical variables) comparing the proportion of patients in the most frequent category between DrotAA vs NonDrotAA, clustering on the matched sets and with weights based on the number of patients in DrotAA and NonDrotAA matched sets. Patients in the unknown categories have been excluded from the tests. No adjustments have been made to account for multiple inference.

1. Demographics for Matched-INDICATED Primary Analysis Population by IRP B Genotype and Treatment

|  | | | | | | |
| --- | --- | --- | --- | --- | --- | --- |
|  | IRP B+ | | IRP B- | | All Genotypes Combined | |
| Demographic Characteristics^a^ | DrotAA (n=148) | NonDrotAA (n=424) | DrotAA (n=486) | DrotAA (n=1239) | NonDrotAA (n=639) | DrotAA (n=1684) |
|  |  |  |  |  |  |  |
| Age |  |  |  |  |  |  |
| Mean | 59.33 | 57.80 | 59.42 | 60.00 | 59.41 | 59.45 |
| SD | 15.362 | 9.612 | 15.220 | 9.247 | 15.190 | 9.305 |
| Min | 21.0 | 18.0 | 20.0 | 18.0 | 20.0 | 18.0 |
| Q1 | 52.0 | 47.0 | 48.0 | 50.0 | 49.0 | 50.0 |
| Median | 61.0 | 59.0 | 60.0 | 61.0 | 60.0 | 60.0 |
| Q3 | 69.0 | 70.0 | 72.0 | 72.0 | 71.0 | 72.0 |
| Max | 98.0 | 90.0 | 90.0 | 96.0 | 98.0 | 96.0 |
| *P*-value^b^ | 0.2347 |  | 0.1491 |  | 0.9441 |  |
|  |  |  |  |  |  |  |
| Gender (%) |  |  |  |  |  |  |
| Male | 58.1 | 61.7 | 60.3 | 60.3 | 59.9 | 60.6 |
| Female | 41.9 | 38.3 | 39.7 | 39.6 | 40.1 | 39.3 |
| Unknown | 0.0 | 0.0 | 0.0 | 0.1 | 0.0 | 0.1 |
| *P*-value^b^ | 0.4220 |  | 0.9648 |  | 0.7401 |  |
|  |  |  |  |  |  |  |
| Recorded Race (%) |  |  |  |  |  |  |
| American Indian or Alaska Native | 0.0 | 0.0 | 0.6 | 0.5 | 0.5 | 0.4 |
| Asian | 2.0 | 1.7 | 1.6 | 1.5 | 1.7 | 1.6 |
| Black or African American | 7.4 | 7.7 | 6.8 | 6.2 | 6.9 | 6.7 |
| White | 77.7 | 75.6 | 77.6 | 78.0 | 77.6 | 77.2 |
| Unknown | 12.8 | 13.8 | 13.2 | 13.8 | 13.1 | 13.8 |
| Other | 0.0) | 1.1 | 0.2 | 0.1 | 0.2 | 0.3 |
| *P*-value^b^ | 0.6748 |  | 0.5692 |  | 0.8900 |  |
|  |  |  |  |  |  |  |
| Recorded Ethnicity (%) |  |  |  |  |  |  |
| Hispanic or Latino | 0.7 | 1.3 | 2.3 | 1.8 | 1.9 | 1.7 |
| Not Hispanic or Latino | 86.5 | 84.3 | 84.6 | 84.3 | 85.0 | 84.3 |
| Unknown | 12.8 | 14.3 | 13.2 | 13.8 | 13.1 | 14.0 |
| *P*-value^b^ | 0.5252 |  | 0.5791 |  | 0.7809 |  |
|  |  |  |  |  |  |  |
| Genetically Determined Continent of Origin (%) |  |  |  |  |  |  |
| European | 81.1 | 82.8 | 81.9 | 82.4 | 81.7 | 82.4 |
| African | 6.8 | 6.0 | 4.7 | 4.7 | 5.2 | 5.0 |
| Asian | 1.4 | 2.2 | 4.3 | 3.2 | 3.6 | 2.9 |
| Other | 10.8 | 9.0 | 9.1 | 9.8 | 9.5 | 9.7 |
| *P*-value^b^ | 0.6726 |  | 0.8331 |  | 0.6606 |  |
|  |  |  |  |  |  |  |

^a^Percentages have been calculated relative to the number of patients in the population. Summary statistics for the NonDrotAA group have been weighted to reflect the unequal numbers of DrotAA and NonDrotAA patients in each of the matched sets.

^b^Descriptive *P*-values are from clustered regression analysis using linear regression (numeric variables) or binary logistic regression (categorical variables) comparing the proportion of patients in the most frequent category between DrotAA vs NonDrotAA, clustering on the matched sets and with weights based on the number of patients in DrotAA and NonDrotAA matched sets. Patients in the unknown categories have been excluded from the tests. No adjustments have been made to account for multiple inference.

1. Baseline Medical History or Comorbidities for Matched-INDICATED Primary Analysis Population by IRP A Genotype and Treatment

| Percentage of Patients | | | | | | |
| --- | --- | --- | --- | --- | --- | --- |
|  | IRP A+ | | IRP A- | | All Genotypes Combined | |
| Preexisting Condition or Comorbidity^a^ | DrotAA (n=197) | NonDrotAA (n=517) | DrotAA (n=440) | NonDrotAA (n=1159) | DrotAA (n=639) | NonDrotAA (n=1684) |
|  |  |  |  |  |  |  |
| Hypertension |  |  |  |  |  |  |
| Yes | 18.3 | 13.9 | 15.2 | 14.0 | 16.1 | 14.0 |
| No | 17.8 | 17.3 | 14.5 | 17.7 | 15.5 | 17.5 |
| Unknown | 64.0 | 68.9 | 70.2 | 68.4 | 68.4 | 68.5 |
| *P*-value^b^ | 0.3792 |  | 0.1232 |  | 0.0741 |  |
|  |  |  |  |  |  |  |
| Myocardial infarction |  |  |  |  |  |  |
| Yes | 6.1 | 3.8 | 2.3 | 4.9 | 3.4 | 4.5 |
| No | 45.2 | 45.0 | 45.7 | 45.2 | 45.4 | 45.0 |
| Unknown | 48.7 | 51.2 | 52.0 | 49.8 | 51.2 | 50.4 |
| *P*-value^b^ | 0.2605 |  | 0.0433 |  | 0.2964 |  |
|  |  |  |  |  |  |  |
| Congestive heart failure |  |  |  |  |  |  |
| Yes | 6.1 | 11.3 | 6.4 | 11.6 | 6.3 | 11.4 |
| No | 65.0 | 63.6 | 66.6 | 63.3 | 65.9 | 63.2 |
| Unknown | 28.9 | 25.2 | 27.0 | 25.1 | 27.9 | 25.3 |
| *P*-value^b^ | 0.0284 |  | 0.0024 |  | 0.0001 |  |
|  |  |  |  |  |  |  |
| COPD |  |  |  |  |  |  |
| Yes | 6.6 | 10.4 | 9.1 | 10.6 | 8.5 | 10.6 |
| No | 62.9 | 62.0 | 62.5 | 61.1 | 62.4 | 61.2 |
| Unknown | 30.5 | 27.7 | 28.4 | 28.3 | 29.1 | 28.2 |
| *P*-value^b^ | 0.1472 |  | 0.4164 |  | 0.1133 |  |
|  |  |  |  |  |  |  |
| Diabetes |  |  |  |  |  |  |
| Yes | 22.8 | 23.5 | 20.5 | 19.6 | 21.1 | 20.7 |
| No | 59.9 | 59.6 | 62.0 | 63.0 | 61.2 | 61.8 |
| Unknown | 17.3 | 16.9 | 17.5 | 17.4 | 17.7 | 17.4 |
| *P*-value^b^ | 0.8693 |  | 0.6613 |  | 0.8061 |  |
|  |  |  |  |  |  |  |
| Pancreatitis |  |  |  |  |  |  |
| Yes | 1.0 | 1.5 | 1.1 | 0.9 | 1.1 | 1.1 |
| No | 23.9 | 21.1 | 19.1 | 21.4 | 20.5 | 21.3 |
| Unknown | 75.1 | 77.4 | 79.8 | 77.6 | 78.4 | 77.6 |
| *P*-value^b^ | 0.5874 |  | 0.6111 |  | 0.9752 |  |
|  |  |  |  |  |  |  |
| Renal dialysis |  |  |  |  |  |  |
| Yes | 4.1 | 3.4 | 2.0 | 4.3 | 2.7 | 4.0 |
| No | 48.2 | 42.8 | 43.0 | 44.5 | 44.4 | 43.9 |
| Unknown | 47.7 | 53.8 | 55.0 | 51.2 | 52.9 | 52.1 |
| *P*-value^b^ | 0.8687 |  | 0.0582 |  | 0.1084 |  |
|  |  |  |  |  |  |  |
| Cirrhosis |  |  |  |  |  |  |
| Yes | 2.5 | 5.4 | 3.0 | 6.0 | 2.8 | 5.8 |
| No | 69.5 | 65.0 | 67.0 | 65.8 | 67.6 | 65.4 |
| Unknown | 27.9 | 29.6 | 30.0 | 28.1 | 29.6 | 28.8 |
| *P*-value^b^ | 0.1049 |  | 0.0189 |  | 0.0049 |  |
|  |  |  |  |  |  |  |
| Other liver disease |  |  |  |  |  |  |
| Yes | 6.1 | 8.2 | 5.2 | 7.2 | 5.5 | 7.5 |
| No | 22.8 | 20.8 | 21.1 | 21.3 | 21.6 | 21.1 |
| Unknown | 71.1 | 71.0 | 73.6 | 71.5 | 72.9 | 71.4 |
| *P*-value^b^ | 0.2286 |  | 0.1106 |  | 0.0078 |  |
|  |  |  |  |  |  |  |
| Malignancy |  |  |  |  |  |  |
| Yes | 13.2 | 16.8 | 14.8 | 17.0 | 14.2 | 16.9 |
| No | 65.5 | 64.6 | 66.1 | 64.6 | 65.9 | 64.6 |
| Unknown | 21.3 | 18.5 | 19.1 | 18.4 | 19.9 | 18.5 |
| *P*-value^b^ | 0.2841 |  | 0.3402 |  | 0.1674 |  |
|  |  |  |  |  |  |  |
| Immunocompromised |  |  |  |  |  |  |
| Yes | 13.2 | 16.8 | 14.8 | 17.0 | 14.2 | 16.9 |
| No | 65.5 | 64.6 | 66.1 | 64.6 | 65.9 | 64.6 |
| Unknown | 21.3 | 18.5 | 19.1 | 18.4 | 19.9 | 18.5 |
| *P*-value^b^ | 0.2841 |  | 0.3402 |  | 0.1674 |  |
|  |  |  |  |  |  |  |

^a^Percentages have been calculated relative to the number of patients in the population. Summary statistics for the NonDrotAA group have been weighted to reflect the unequal numbers of DrotAA and NonDrotAA patients in each of the matched sets.

^b^Descriptive *P*-values are from clustered binary logistic regression comparing the proportion of patients in the most frequent category between DrotAA vs NonDrotAA, clustering on the matched sets and with weights based on the number of patients in DrotAA and NonDrotAA matched sets. Patients in the unknown categories have been excluded from the tests. No adjustments have been made to account for multiple inference.

1. Baseline Medical History or Comorbidities for Matched-INDICATED Primary Analysis Population by IRP B Genotype and Treatment

| Percentage of Patients | | | | | | |
| --- | --- | --- | --- | --- | --- | --- |
|  | IRP B+ | | IRP B- | | All Genotypes Combined | |
| Preexisting Condition or Comorbidity^a^ | DrotAA (n=148) | NonDrotAA (n=424) | DrotAA (n=486) | NonDrotAA (n=1239) | DrotAA (n=639) | NonDrotAA (n=1684) |
|  |  |  |  |  |  |  |
| Hypertension |  |  |  |  |  |  |
| Yes | 15.5 | 14.0 | 16.5 | 14.0 | 16.1 | 14.0 |
| No | 16.9 | 18.1 | 15.0 | 17.3 | 15.5 | 17.5 |
| Unknown | 67.6 | 67.8 | 68.5 | 68.7 | 68.4 | 68.5 |
| *P*-value^b^ | 0.5634 |  | 0.0865 |  | 0.0741 |  |
|  |  |  |  |  |  |  |
| Myocardial infarction |  |  |  |  |  |  |
| Yes | 4.7 | 4.2 | 3.1 | 4.6 | 3.4 | 4.5 |
| No | 52.7 | 46.1 | 43.2 | 44.8 | 45.4 | 45.0 |
| Unknown | 42.6 | 49.7 | 53.7 | 50.6 | 51.2 | 50.4 |
| *P*-value^b^ | 0.9640 |  | 0.2585 |  | 0.2964 |  |
|  |  |  |  |  |  |  |
| Congestive heart failure |  |  |  |  |  |  |
| Yes | 5.4 | 11.1 | 6.6 | 11.6 | 6.3 | 11.4 |
| No | 67.6 | 63.7 | 65.0 | 62.7 | 65.9 | 63.2 |
| Unknown | 27.0 | 25.2 | 28.4 | 25.7 | 27.9 | 25.3 |
| *P*-value^b^ | 0.0265 |  | 0.0015 |  | 0.0001 |  |
|  |  |  |  |  |  |  |
| COPD |  |  |  |  |  |  |
| Yes | 12.2 | 8.5 | 7.4 | 11.4 | 8.5 | 10.6 |
| No | 60.1 | 63.4 | 62.8 | 60.1 | 62.4 | 61.2 |
| Unknown | 27.7 | 28.1 | 29.8 | 28.5 | 29.1 | 28.2 |
| *P*-value^b^ | 0.1409 |  | 0.0132 |  | 0.1133 |  |
|  |  |  |  |  |  |  |
| Diabetes |  |  |  |  |  |  |
| Yes | 16.9 | 19.4 | 22.4 | 21.4 | 21.1 | 20.7 |
| No | 65.5 | 62.4 | 59.7 | 61.2 | 61.2 | 61.8 |
| Unknown | 17.6 | 18.2 | 17.9 | 17.4 | 17.7 | 17.4 |
| *P*-value^b^ | 0.4753 |  | 0.6022 |  | 0.8061 |  |
|  |  |  |  |  |  |  |
| Pancreatitis |  |  |  |  |  |  |
| Yes | 0.7 | 0.6 | 1.2 | 1.3 | 1.1 | 1.1 |
| No | 20.9 | 22.1 | 20.4 | 21.1 | 20.5 | 21.3 |
| Unknown | 78.4 | 77.3 | 78.4 | 77.7 | 78.4 | 77.6 |
| *P*-value^b^ | 0.9124 |  | 0.9999 |  | 0.9752 |  |
|  |  |  |  |  |  |  |
| Renal dialysis |  |  |  |  |  |  |
| Yes | 1.4 | 2.8 | 3.1 | 4.5 | 2.7 | 4.0 |
| No | 53.4 | 42.4 | 42.0 | 44.7 | 44.4 | 43.9 |
| Unknown | 45.3 | 54.8 | 54.9 | 50.9 | 52.9 | 52.1 |
| *P*-value^b^ | 0.2303 |  | 0.3280 |  | 0.1084 |  |
|  |  |  |  |  |  |  |
| Cirrhosis |  |  |  |  |  |  |
| Yes | 2.7 | 6.8 | 2.7 | 5.5 | 2.8 | 5.8 |
| No | 68.9 | 61.7 | 67.3 | 66.6 | 67.6 | 65.4 |
| Unknown | 28.4 | 31.6 | 30.0 | 27.9 | 29.6 | 28.8 |
| *P*-value^b^ | 0.0598 |  | 0.0251 |  | 0.0049 |  |
|  |  |  |  |  |  |  |
| Other liver disease |  |  |  |  |  |  |
| Yes | 5.4 | 10.5 | 5.3 | 6.4 | 5.5 | 7.5 |
| No | 20.9 | 20.0 | 21.8 | 21.5 | 21.6 | 21.1 |
| Unknown | 73.6 | 69.6 | 72.8 | 72.2 | 72.9 | 71.4 |
| *P*-value^b^ | 0.1318 |  | 0.1316 |  | 0.0078 |  |
|  |  |  |  |  |  |  |
| Malignancy |  |  |  |  |  |  |
| Yes | 12.8 | 16.0 | 14.8 | 17.1 | 14.2 | 16.9 |
| No | 72.3 | 63.8 | 63.6 | 64.8 | 65.9 | 64.6 |
| Unknown | 14.9 | 20.2 | 21.6 | 18.1 | 19.9 | 18.5 |
| *P*-value^b^ | 0.2170 |  | 0.4500 |  | 0.1674 |  |
|  |  |  |  |  |  |  |
| Immunocompromised |  |  |  |  |  |  |
| Yes | 12.8 | 16.0 | 14.8 | 17.1 | 14.2 | 16.9 |
| No | 72.3 | 63.8 | 63.6 | 64.8 | 65.9 | 64.6 |
| Unknown | 14.9 | 20.2 | 21.6 | 18.1 | 19.9 | 18.5 |
| *P*-value^b^ | 0.2170 |  | 0.4500 |  | 0.1674 |  |
|  |  |  |  |  |  |  |

^a^Percentages have been calculated relative to the number of patients in the population. Summary statistics for the NonDrotAA group have been weighted to reflect the unequal numbers of DrotAA and NonDrotAA patients in each of the matched sets.

^b^Descriptive *P*-values are from clustered binary logistic regression comparing the proportion of patients in the most frequent category between DrotAA vs NonDrotAA, clustering on the matched sets and with weights based on the number of patients in DrotAA and NonDrotAA matched sets. Patients in the unknown categories have been excluded from the tests. No adjustments have been made to account for multiple inference.

1. Baseline Infection Characteristics for Matched-INDICATED Primary Analysis Population by IRP A Genotype and Treatment

| Percentage of Patients | | | | | | |
| --- | --- | --- | --- | --- | --- | --- |
|  | IRP A+ | | IRP A- | | All Genotypes Combined | |
| Baseline Infection Parameters^a^ | DrotAA (n=197) | NonDrotAA (n=517) | DrotAA (n=440) | NonDrotAA (n=1159) | DrotAA (n=639) | NonDrotAA (n=1684) |
|  |  |  |  |  |  |  |
| Proven or Suspected Infection |  |  |  |  |  |  |
| Proven | 82.2 | 67.8 | 74.1 | 68.2 | 76.5 | 68.0 |
| Suspected | 12.7 | 25.9 | 20.7 | 25.7 | 18.2 | 25.7 |
| Unknown | 5.1 | 6.3 | 5.2 | 6.1 | 5.3 | 6.3 |
| *P*-value^b^ | <.0001 |  | 0.0076 |  | <.0001 |  |
|  |  |  |  |  |  |  |
| Proven Infection^c^ | n=162 | n=351 | n=326 | n=790 | n=489 | n=1145 |
| Gram- | 25.9 | 30.6 | 32.8 | 33.5 | 30.5 | 32.6 |
| *P*-value^b^ | 0.3378 |  | 0.8522 |  | 0.4063 |  |
| Gram+ | 44.4 | 35.8 | 37.4 | 37.6 | 39.7 | 36.9 |
| *P*-value^b^ | 0.0471 |  | 0.9936 |  | 0.2120 |  |
| Gram Variable | 0.0 | 0.2 | 0.6 | 0.0 | 0.4 | 0.1 |
| *P*-value^b^ |  |  |  |  | 0.1660 |  |
| Fungi | 3.7 | 6.0 | 2.1 | 5.7 | 2.7 | 5.8 |
| *P*-value^b^ | 0.2977 |  | 0.0142 |  | 0.0049 |  |
| Virus | 3.1 | 2.0 | 2.8 | 2.1 | 2.9 | 2.1 |
| *P*-value^b^ | 0.4386 |  | 0.4945 |  | 0.3040 |  |
| Unknown | 13.0 | 14.0 | 9.5 | 15.5 | 10.6 | 15.0 |
| *P*-value^b^ | 0.6808 |  | 0.0029 |  | 0.0028 |  |
| Other | 0.0 | 0.5 | 0.0 | 0.9 | 0.0 | 0.8 |
| *P*-value^b^ |  |  |  |  |  |  |
|  |  |  |  |  |  |  |
| Suspected Infection | n=25 | n=134 | n=91 | n=298 | n=116 | n=433 |
| Culture requested but No IV Antibiotics given | 4.0 | 5.8 | 12.1 | 6.9 | 10.3 | 6.6 |
| IV Antibiotics given but No Culture requested | 72.0 | 76.9 | 71.4 | 71.5 | 71.6 | 73.2 |
| Both Culture requested and IV Antibiotics given | 24.0 | 17.2 | 16.5 | 21.6 | 18.1 | 20.2 |
| *P*-value^b^ | 0.3716 |  | 0.3383 |  | 0.5858 |  |
|  |  |  |  |  |  |  |
| Origin of Sepsis |  |  |  |  |  |  |
| Nosocomial | 6.1 | 15.0 | 8.4 | 12.3 | 7.7 | 13.1 |
| Community acquired | 38.1 | 34.8 | 42.7 | 36.3 | 41.2 | 35.7 |
| Unknown | 55.8 | 50.2 | 48.9 | 51.4 | 51.2 | 51.2 |
| *P*-value^b^ | 0.0022 |  | 0.0051 |  | <.0001 |  |
|  |  |  |  |  |  |  |
| Anatomic Site of Primary Infection |  |  |  |  |  |  |
| Lung | 53.3 | 48.1 | 54.5 | 48.6 | 54.0 | 48.3 |
| Abdomen | 14.2 | 12.5 | 13.4 | 12.9 | 13.6 | 12.8 |
| CNS | 2.0 | 1.2 | 0.5 | 0.4 | 0.9 | 0.7 |
| Blood | 3.6 | 3.1 | 2.7 | 3.5 | 3.0 | 3.3 |
| Urinary tract | 2.0 | 3.7 | 4.8 | 3.4 | 3.9 | 3.4 |
| Unknown | 19.8 | 26.6 | 18.9 | 26.8 | 19.4 | 26.9 |
| Other | 5.1 | 4.9 | 5.2 | 4.3 | 5.2 | 4.5 |
| *P*-value^b^ | 0.9955 |  | 0.7419 |  | 0.6815 |  |
|  |  |  |  |  |  |  |

^a^Percentages have been calculated relative to the number of patients in the population, with the exception of Proven Infection types (Gram +/-, etc) and Suspected Infection evidence (culture requested, etc), where the numbers of patients with proven or suspected infections have been used, respectively. Summary statistics for the NonDrotAA group have been weighted to reflect the unequal numbers of DrotAA and NonDrotAA patients in each of the matched sets.

^b^Descriptive *P*-values are from clustered binary logistic regression comparing the proportion of patients in the most frequent category between DrotAA vs NonDrotAA, clustering on the matched sets and with weights based on the number of patients in DrotAA and NonDrotAA matched sets. Patients in the unknown categories have been excluded from the tests. No adjustments have been made to account for multiple inference.

^c^Not mutually exclusive categories.

1. Baseline Infection Characteristics for Matched-INDICATED Primary Analysis Population by IRP B Genotype and Treatment

| Percentage of Patients | | | | | | |
| --- | --- | --- | --- | --- | --- | --- |
|  | IRP B+ | | IRP B- | | All Genotypes Combined | |
| Baseline Infection Parameters^a^ | DrotAA (n=148) | NonDrotAA (n=424) | DrotAA (n=486) | NonDrotAA (n=1239) | DrotAA (n=639) | NonDrotAA (n=1684) |
|  |  |  |  |  |  |  |
| Proven or Suspected Infection |  |  |  |  |  |  |
| Proven | 74.3 | 64.9 | 77.6 | 69.0 | 76.5 | 68.0 |
| Suspected | 20.9 | 30.2 | 16.9 | 24.2 | 18.2 | 25.7 |
| Unknown | 4.7 | 4.9 | 5.6 | 6.8 | 5.3 | 6.3 |
| *P*-value^b^ | 0.0251 |  | <.0001 |  | <.0001 |  |
|  |  |  |  |  |  |  |
| Proven Infection^c^ | n=110 | n=275 | n=377 | n=855 | n=489 | n=1145 |
| Gram- | 23.6 | 32.7 | 32.1 | 31.9 | 30.5 | 32.6 |
| *P*-value^b^ | 0.1057 |  | 0.9864 |  | 0.4063 |  |
| Gram+ | 49.1 | 35.4 | 36.9 | 37.8 | 39.7 | 36.9 |
| *P*-value^b^ | 0.0120 |  | 0.7326 |  | 0.2120 |  |
| Gram Variable | 0.0 | 0.0 | 0.5 | 0.1 | 0.4 | 0.1 |
| *P*-value^b^ | . |  | 0.1720 |  | 0.1660 |  |
| Fungi | 1.8 | 6.5 | 2.9 | 5.7 | 2.7 | 5.8 |
| *P*-value^b^ | 0.0852 |  | 0.0371 |  | 0.0049 |  |
| Virus | 4.5 | 3.3 | 2.4 | 1.7 | 2.9 | 2.1 |
| *P*-value^b^ | 0.5325 |  | 0.3930 |  | 0.3040 |  |
| Unknown | 10.9 | 14.8 | 10.6 | 15.2 | 10.6 | 15.0 |
| *P*-value^b^ | 0.2483 |  | 0.0111 |  | 0.0028 |  |
| Other | 0.0 | 1.1 | 0.0 | 0.7 | 0.0 | 0.8 |
| *P*-value^b^ |  |  |  |  |  |  |
|  |  |  |  |  |  |  |
| Suspected Infection | n=31 | n=128 | n=82 | n=300 | n=116 | n=433 |
| Culture requested but No IV Antibiotics given | 3.2 | 5.4 | 12.2 | 7.3 | 10.3 | 6.6 |
| IV Antibiotics given but No Culture requested | 90.3 | 74.2 | 65.9 | 72.8 | 71.6 | 73.2 |
| Both Culture requested and IV Antibiotics given | 6.5 | 20.3 | 22.0 | 19.9 | 18.1 | 20.2 |
| *P*-value^b^ | 0.0930 |  | 0.7211 |  | 0.5858 |  |
|  |  |  |  |  |  |  |
| Origin of Sepsis |  |  |  |  |  |  |
| Nosocomial | 7.4 | 13.3 | 7.8 | 12.9 | 7.7 | 13.1 |
| Community acquired | 41.9 | 35.7 | 40.5 | 35.6 | 41.2 | 35.7 |
| Unknown | 50.7 | 51.0 | 51.6 | 51.6 | 51.2 | 51.2 |
| *P*-value^b^ | 0.0374 |  | 0.0002 |  | <.0001 |  |
|  |  |  |  |  |  |  |
| Anatomic Site of Primary Infection |  |  |  |  |  |  |
| Lung | 52.0 | 49.3 | 54.5 | 48.5 | 54.0 | 48.3 |
| Abdomen | 15.5 | 12.0 | 13.2 | 12.6 | 13.6 | 12.8 |
| CNS | 0.0 | 0.7 | 1.2 | 0.6 | 0.9 | 0.7 |
| Blood | 3.4 | 2.7 | 2.7 | 3.5 | 3.0 | 3.3 |
| Urinary tract | 5.4 | 3.8 | 3.3 | 3.3 | 3.9 | 3.4 |
| Unknown | 18.2 | 27.9 | 20.0 | 26.8 | 19.4 | 26.9 |
| Other | 5.4 | 3.7 | 5.1 | 4.8 | 5.2 | 4.5 |
| *P*-value^b^ | 0.2872 |  | 0.4292 |  | 0.6815 |  |
|  |  |  |  |  |  |  |

^a^Percentages have been calculated relative to the number of patients in the population, with the exception of Proven Infection types (Gram +/-, etc) and Suspected Infection evidence (culture requested, etc), where the numbers of patients with proven or suspected infections have been used, respectively. Summary statistics for the NonDrotAA group have been weighted to reflect the unequal numbers of DrotAA and NonDrotAA patients in each of the matched sets.

^b^Descriptive *P*-values are from clustered binary logistic regression comparing the proportion of patients in the most frequent category between DrotAA vs NonDrotAA, clustering on the matched sets and with weights based on the number of patients in DrotAA and NonDrotAA matched sets. Patients in the unknown categories have been excluded from the tests. No adjustments have been made to account for multiple inference.

^c^Not mutually exclusive categories.

1. Baseline Organ Dysfunctions for Matched-INDICATED Primary Analysis Population by IRP A Genotype and Treatment

|  | | | | | | |
| --- | --- | --- | --- | --- | --- | --- |
|  | IRP A+ | | IRP A- | | All Genotypes Combined | |
| Baseline Organ Dysfunction Parameter^a^ | DrotAA (n=197) | NonDrotAA (n=517) | DrotAA (n=440) | NonDrotAA (n=1159) | DrotAA (n=639) | NonDrotAA (n=1684) |
|  |  |  |  |  |  |  |
| Number of Organ Systems with Dysfunction (%) |  |  |  |  |  |  |
| 1 | 0.0 | 1.2 | 0.0 | 0.5 | 0.0 | 0.7 |
| 2 | 21.8 | 22.5 | 19.8 | 21.1 | 20.7 | 21.6 |
| 3 | 36.5 | 34.6 | 35.2 | 34.4 | 35.5 | 34.5 |
| 4 | 24.9 | 26.3 | 27.3 | 30.0 | 26.4 | 28.9 |
| 5 | 14.7 | 12.7 | 13.9 | 11.9 | 14.1 | 12.1 |
| 6 | 2.0 | 2.7 | 3.9 | 2.1 | 3.3 | 2.3 |
| *P*-value^b^ | 0.9427 |  | 0.6778 |  | 0.8154 |  |
|  |  |  |  |  |  |  |
| Number of Organ Systems with Dysfunction |  |  |  |  |  |  |
| Mean | 3.39 | 3.35 | 3.47 | 3.38 | 3.44 | 3.37 |
| SD | 1.047 | 0.668 | 1.075 | 0.631 | 1.068 | 0.642 |
| Min | 2.0 | 1.0 | 2.0 | 1.0 | 2.0 | 1.0 |
| Q1 | 3.0 | 3.0 | 3.0 | 3.0 | 3.0 | 3.0 |
| Median | 3.0 | 3.0 | 3.0 | 3.0 | 3.0 | 3.0 |
| Q3 | 4.0 | 4.0 | 4.0 | 4.0 | 4.0 | 4.0 |
| Max | 6.0 | 6.0 | 6.0 | 6.0 | 6.0 | 6.0 |
| *P*-value^b^ | 0.6874 |  | 0.0582 |  | 0.0451 |  |
|  |  |  |  |  |  |  |
| Cardiovascular (%) |  |  |  |  |  |  |
| Yes | 98.5 | 97.5 | 98.9 | 98.4 | 98.7 | 98.1 |
| No | 1.5 | 1.7 | 0.9 | 0.8 | 1.1 | 1.1 |
| Unknown | 0.0 | 0.8 | 0.2 | 0.8 | 0.2 | 0.8 |
| *P*-value^b^ | 0.7105 |  | 0.8738 |  | 0.5879 |  |
|  |  |  |  |  |  |  |
|  |  |  |  |  |  |  |
| Pulmonary (%) |  |  |  |  |  |  |
| Yes | 97.0 | 95.3 | 95.5 | 95.7 | 95.9 | 95.5 |
| No | 3.0 | 3.8 | 3.9 | 3.2 | 3.6 | 3.4 |
| Unknown | 0.0 | 0.9 | 0.7 | 1.2 | 0.5 | 1.1 |
| *P*-value^b^ | 0.5350 |  | 0.3663 |  | 0.9496 |  |
|  |  |  |  |  |  |  |
| CNS (%) |  |  |  |  |  |  |
| Yes | 26.4 | 31.0 | 27.5 | 31.4 | 27.1 | 31.3 |
| No | 39.6 | 30.8 | 31.1 | 30.3 | 33.8 | 30.4 |
| Unknown | 34.0 | 38.2 | 41.4 | 38.3 | 39.1 | 38.3 |
| *P*-value^b^ | 0.0258 |  | 0.1826 |  | 0.0040 |  |
|  |  |  |  |  |  |  |
| Coagulation (%) |  |  |  |  |  |  |
| Yes | 31.5 | 26.3 | 31.8 | 25.4 | 31.6 | 25.6 |
| No | 67.5 | 71.9 | 67.3 | 73.3 | 67.3 | 72.8 |
| Unknown | 1.0 | 1.8 | 0.9 | 1.3 | 1.1 | 1.6 |
| *P*-value^b^ | 0.1783 |  | 0.0014 |  | 0.0002 |  |
|  |  |  |  |  |  |  |
| Renal (%) |  |  |  |  |  |  |
| Yes | 62.4 | 61.4 | 68.0 | 62.7 | 66.0 | 62.1 |
| No | 36.0 | 37.7 | 31.1 | 36.5 | 32.7 | 37.0 |
| Unknown | 1.5 | 0.8 | 0.9 | 0.8 | 1.3 | 0.9 |
| *P*-value^b^ | 0.7002 |  | 0.0096 |  | 0.0026 |  |
|  |  |  |  |  |  |  |
| Hepatic (%) |  |  |  |  |  |  |
| Yes | 22.8 | 23.5 | 25.2 | 24.7 | 24.4 | 24.4 |
| No | 69.0 | 69.4 | 68.6 | 68.3 | 68.7 | 68.4 |
| Unknown | 8.1 | 7.2 | 6.1 | 7.1 | 6.9 | 7.2 |
| *P*-value^b^ | 0.8717 |  | 0.8944 |  | 0.9613 |  |
|  |  |  |  |  |  |  |

^a^Percentages have been calculated relative to the number of patients in the population. Summary statistics for the NonDrotAA group have been weighted to reflect the unequal numbers of DrotAA and NonDrotAA patients in each of the matched sets.

^b^Descriptive *P*-values are from clustered regression analysis using linear regression (numeric variables) or binary logistic regression (categorical variables) comparing the proportion of patients in the most frequent category between DrotAA vs NonDrotAA, clustering on the matched sets and with weights based on the number of patients in DrotAA and NonDrotAA matched sets. For Number of Organ Systems with Dysfunction, the distribution was split at the modal count into higher versus lower counts. Patients in the unknown categories have been excluded from the tests. No adjustments have been made to account for multiple inference.

1. Baseline Organ Dysfunctions for Matched-INDICATED Primary Analysis Population by IRP B Genotype and Treatment

|  | | | | | | |
| --- | --- | --- | --- | --- | --- | --- |
|  | IRP B+ | | IRP B- | | All Genotypes Combined | |
| Baseline Organ Dysfunction Parameter^a^ | DrotAA (n=148) | NonDrotAA (n=424) | DrotAA (n=486) | NonDrotAA (n=1239) | DrotAA (n=639) | NonDrotAA (n=1684) |
|  |  |  |  |  |  |  |
| Number of Organ Systems with Dysfunction (%) |  |  |  |  |  |  |
| 1 | 0.0 | 0.7 | 0.0 | 0.7 | 0.0 | 0.7 |
| 2 | 18.9 | 21.5 | 21.2 | 21.9 | 20.7 | 21.6 |
| 3 | 33.1 | 33.8 | 36.2 | 34.7 | 35.5 | 34.5 |
| 4 | 29.7 | 27.3 | 25.5 | 29.2 | 26.4 | 28.9 |
| 5 | 14.9 | 14.0 | 13.8 | 11.4 | 14.1 | 12.1 |
| 6 | 3.4 | 2.7 | 3.3 | 2.2 | 3.3 | 2.3 |
| *P*-value^b^ | 0.3467 |  | 0.8985 |  | 0.8154 |  |
|  |  |  |  |  |  |  |
| Number of Organ Systems with Dysfunction |  |  |  |  |  |  |
| Mean | 3.51 | 3.40 | 3.42 | 3.35 | 3.44 | 3.37 |
| SD | 1.066 | 0.666 | 1.069 | 0.637 | 1.068 | 0.642 |
| Min | 2.0 | 1.0 | 2.0 | 1.0 | 2.0 | 1.0 |
| Q1 | 3.0 | 3.0 | 3.0 | 3.0 | 3.0 | 3.0 |
| Median | 3.0 | 3.0 | 3.0 | 3.0 | 3.0 | 3.0 |
| Q3 | 4.0 | 4.0 | 4.0 | 4.0 | 4.0 | 4.0 |
| Max | 6.0 | 6.0 | 6.0 | 6.0 | 6.0 | 6.0 |
| *P*-value^b^ | 0.2570 |  | 0.1331 |  | 0.0451 |  |
|  |  |  |  |  |  |  |
| Cardiovascular (%) |  |  |  |  |  |  |
| Yes | 98.6 | 98.6 | 98.8 | 97.9 | 98.7 | 98.1 |
| No | 0.7 | 0.5 | 1.2 | 1.3 | 1.1 | 1.1 |
| Unknown | 0.7 | 0.9 | 0.0 | 0.7 | 0.2 | 0.8 |
| *P*-value^b^ | 0.8121 |  | 0.9529 |  | 0.5879 |  |
|  |  |  |  |  |  |  |
| Pulmonary (%) |  |  |  |  |  |  |
| Yes | 96.6 | 96.5 | 95.9 | 95.2 | 95.9 | 95.5 |
| No | 3.4 | 3.0 | 3.5 | 3.5 | 3.6 | 3.4 |
| Unknown | 0.0 | 0.5 | 0.6 | 1.3 | 0.5 | 1.1 |
| *P*-value^b^ | 0.8737 |  | 0.8347 |  | 0.9496 |  |
|  |  |  |  |  |  |  |
| CNS (%) |  |  |  |  |  |  |
| Yes | 27.0 | 31.1 | 27.4 | 31.7 | 27.1 | 31.3 |
| No | 38.5 | 32.1 | 32.5 | 29.9 | 33.8 | 30.4 |
| Unknown | 34.5 | 36.8 | 40.1 | 38.4 | 39.1 | 38.3 |
| *P*-value^b^ | 0.1235 |  | 0.0437 |  | 0.0040 |  |
|  |  |  |  |  |  |  |
| Coagulation (%) |  |  |  |  |  |  |
| Yes | 34.5 | 26.8 | 30.5 | 24.9 | 31.6 | 25.6 |
| No | 63.5 | 71.4 | 68.7 | 73.7 | 67.3 | 72.8 |
| Unknown | 2.0 | 1.7 | 0.8 | 1.4 | 1.1 | 1.6 |
| *P*-value^b^ | 0.0569 |  | 0.0073 |  | 0.0002 |  |
|  |  |  |  |  |  |  |
| Renal (%) |  |  |  |  |  |  |
| Yes | 69.6 | 60.7 | 64.8 | 62.4 | 66.0 | 62.1 |
| No | 28.4 | 38.5 | 34.2 | 36.6 | 32.7 | 37.0 |
| Unknown | 2.0 | 0.8 | 1.0 | 1.0 | 1.3 | 0.9 |
| *P*-value^b^ | 0.0121 |  | 0.1831 |  | 0.0026 |  |
|  |  |  |  |  |  |  |
| Hepatic (%) |  |  |  |  |  |  |
| Yes | 24.3 | 26.6 | 24.5 | 23.3 | 24.4 | 24.4 |
| No | 68.9 | 66.2 | 68.5 | 69.6 | 68.7 | 68.4 |
| Unknown | 6.8 | 7.2 | 7.0 | 7.1 | 6.9 | 7.2 |
| *P*-value^b^ | 0.5474 |  | 0.5824 |  | 0.9613 |  |
|  |  |  |  |  |  |  |

^a^Percentages have been calculated relative to the number of patients in the population. Summary statistics for the NonDrotAA group have been weighted to reflect the unequal numbers of DrotAA and NonDrotAA patients in each of the matched sets.

^b^Descriptive *P*-values are from clustered regression analysis using linear regression (numeric variables) or binary logistic regression (categorical variables) comparing the proportion of patients in the most frequent category between DrotAA vs NonDrotAA, clustering on the matched sets and with weights based on the number of patients in DrotAA and NonDrotAA matched sets. For Number of Organ Systems with Dysfunction, the distribution was split at the modal count into higher versus lower counts. Patients in the unknown categories have been excluded from the tests. No adjustments have been made to account for multiple inference.

1. Baseline Risk of Death Characterization for Matched-INDICATED Primary Analysis Population by IRP A Genotype and Treatment

|  | IRP A+ | | IRP A- | | All Genotypes Combined | |
| --- | --- | --- | --- | --- | --- | --- |
| Baseline Risk of Death Parameter^a^ | DrotAA (n=197) | NonDrotAA (n=517) | DrotAA (n=440) | NonDrotAA (n=1159) | DrotAA (n=639) | NonDrotAA (n=1684) |
|  |  |  |  |  |  |  |
| APACHE II |  |  |  |  |  |  |
| N | 141 | 354 | 296 | 802 | 438 | 1160 |
| Mean | 25.14 | 26.35 | 26.14 | 25.19 | 25.80 | 25.50 |
| SD | 7.349 | 5.272 | 8.146 | 4.730 | 7.897 | 4.922 |
| Min | 8.0 | 5.0 | 8.0 | 1.0 | 8.0 | 1.0 |
| Q1 | 20.0 | 20.0 | 20.0 | 20.0 | 20.0 | 20.0 |
| Median | 24.0 | 25.0 | 26.0 | 25.0 | 25.0 | 25.0 |
| Q3 | 30.0 | 33.0 | 31.5 | 30.0 | 31.0 | 31.0 |
| Max | 43.0 | 48.0 | 47.0 | 46.0 | 47.0 | 48.0 |
| *P*-value^b^ | 0.0889 |  | 0.0105 |  | 0.0187 |  |
|  |  |  |  |  |  |  |
| SAPS II |  |  |  |  |  |  |
| N | 116 | 319 | 270 | 696 | 388 | 1023 |
| Mean | 59.73 | 59.31 | 59.26 | 58.44 | 59.36 | 58.68 |
| SD | 20.385 | 11.426 | 18.445 | 11.335 | 19.003 | 11.342 |
| Min | 21.0 | 19.0 | 20.0 | 18.0 | 20.0 | 18.0 |
| Q1 | 44.0 | 46.0 | 46.0 | 44.0 | 44.0 | 44.0 |
| Median | 57.0 | 59.0 | 59.5 | 58.0 | 59.0 | 58.0 |
| Q3 | 74.0 | 71.0 | 71.0 | 69.0 | 72.0 | 70.0 |
| Max | 111.0 | 111.0 | 105.0 | 111.0 | 111.0 | 111.0 |
| *P*-value^b^ | 0.8996 |  | 0.9004 |  | 0.8119 |  |
|  |  |  |  |  |  |  |
| High Risk of Death Criteria Met (%) |  |  |  |  |  |  |
| APACHE II or SAPS II only | 0.0 | 1.2 | 0.0 | 0.6 | 0.0 | 0.8 |
| MOD only | 44.7 | 39.1 | 38.2 | 42.0 | 40.2 | 41.2 |
| Both | 55.3 | 59.7 | 61.8 | 57.4 | 59.8 | 58.0 |
| *P*-value^b^ | 0.2410 |  | 0.0799 |  | 0.2348 |  |
|  |  |  |  |  |  |  |

^a^Percentages have been calculated relative to the number of patients in the population. Summary statistics for the NonDrotAA group have been weighted to reflect the unequal numbers of DrotAA and NonDrotAA patients in each of the matched sets.

^b^Descriptive *P*-values are from clustered regression analysis using linear regression (numeric variables) or binary logistic regression (categorical variables) comparing the proportion of patients in the most frequent category between DrotAA vs NonDrotAA, clustering on the matched sets and with weights based on the number of patients in DrotAA and NonDrotAA matched sets. Patients in the unknown categories have been excluded from the tests. No adjustments have been made to account for multiple inference.

1. Baseline Risk of Death Characterization for Matched-INDICATED Primary Analysis Population by IRP B Genotype and Treatment

|  | | | | | | |
| --- | --- | --- | --- | --- | --- | --- |
|  | IRP B+ | | IRP B- | | All Genotypes Combined | |
| Baseline Risk of Death Parameter^a^ | DrotAA (n=148) | NonDrotAA (n=424) | DrotAA (n=486) | NonDrotAA (n=1239) | DrotAA (n=639) | NonDrotAA (n=1684) |
|  |  |  |  |  |  |  |
| APACHE II |  |  |  |  |  |  |
| N | 110 | 294 | 326 | 856 | 438 | 1160 |
| Mean | 25.25 | 25.49 | 25.97 | 25.48 | 25.80 | 25.50 |
| SD | 7.780 | 5.320 | 7.959 | 4.811 | 7.897 | 4.922 |
| Min | 8.0 | 6.0 | 8.0 | 1.0 | 8.0 | 1.0 |
| Q1 | 20.0 | 19.0 | 20.0 | 20.0 | 20.0 | 20.0 |
| Median | 25.0 | 25.0 | 25.0 | 25.0 | 25.0 | 25.0 |
| Q3 | 31.0 | 32.0 | 31.0 | 31.0 | 31.0 | 31.0 |
| Max | 44.0 | 48.0 | 47.0 | 48.0 | 47.0 | 48.0 |
| *P*-value^b^ | 0.8437 |  | 0.1116 |  | 0.0187 |  |
|  |  |  |  |  |  |  |
| SAPS II |  |  |  |  |  |  |
| N | 77 | 260 | 308 | 750 | 388 | 1023 |
| Mean | 57.78 | 58.67 | 59.81 | 58.74 | 59.36 | 58.68 |
| SD | 17.647 | 11.630 | 19.364 | 11.287 | 19.003 | 11.342 |
| Min | 22.0 | 19.0 | 20.0 | 18.0 | 20.0 | 18.0 |
| Q1 | 45.0 | 44.0 | 44.5 | 45.0 | 44.0 | 44.0 |
| Median | 55.0 | 57.0 | 59.0 | 58.0 | 59.0 | 58.0 |
| Q3 | 70.0 | 70.0 | 73.0 | 70.0 | 72.0 | 70.0 |
| Max | 105.0 | 111.0 | 111.0 | 111.0 | 111.0 | 111.0 |
| *P*-value^b^ | 0.5335 |  | 0.6114 |  | 0.8119 |  |
|  |  |  |  |  |  |  |
| High Risk of Death Criteria Met (%) |  |  |  |  |  |  |
| APACHE II or SAPS II only | 0.0 | 0.7 | 0.0 | 0.8 | 0.0 | 0.8 |
| MOD only | 45.9 | 40.8 | 38.5 | 41.3 | 40.2 | 41.2 |
| Both | 54.1 | 58.5 | 61.5 | 57.9 | 59.8 | 58.0 |
| *P*-value^b^ | 0.3331 |  | 0.0962 |  | 0.2348 |  |
|  |  |  |  |  |  |  |

^a^Percentages have been calculated relative to the number of patients in the population. Summary statistics for the NonDrotAA group have been weighted to reflect the unequal numbers of DrotAA and NonDrotAA patients in each of the matched sets.

^b^Descriptive *P*-values are from clustered regression analysis using linear regression (numeric variables) or binary logistic regression (categorical variables) comparing the proportion of patients in the most frequent category between DrotAA vs NonDrotAA, clustering on the matched sets and with weights based on the number of patients in DrotAA and NonDrotAA matched sets. Patients in the unknown categories have been excluded from the tests. No adjustments have been made to account for multiple inference.

1. Use of Vasopressors and Mechanical Ventilation in Matched-INDICATED Primary Analysis Population by IRP A Genotype and Treatment

Percentage of Patients

|  | IRP A+ | | IRP A- | | All Genotypes Combined | |
| --- | --- | --- | --- | --- | --- | --- |
| Supportive Treatment^a^ | DrotAA (n=197) | NonDrotAA (n=517) | DrotAA (n=440) | NonDrotAA (n=1159) | DrotAA (n=639) | NonDrotAA (n=1684) |
|  |  |  |  |  |  |  |
| Use of Vasopressors |  |  |  |  |  |  |
| Yes | 90.4 | 81.4 | 93.2 | 80.9 | 92.3 | 80.9 |
| No | 9.1 | 17.3 | 6.4 | 18.1 | 7.2 | 18.0 |
| Unknown | 0.5 | 1.3 | 0.5 | 1.0 | 0.5 | 1.1 |
| *P*-value^b^ | 0.0040 |  | <.0001 |  | <.0001 |  |
|  |  |  |  |  |  |  |
| Mechanical Ventilation |  |  |  |  |  |  |
| Yes | 75.6 | 76.2 | 75.7 | 76.0 | 75.7 | 76.2 |
| No | 5.6 | 5.5 | 5.9 | 5.0 | 5.8 | 5.1 |
| Unknown | 18.8 | 18.4 | 18.4 | 18.9 | 18.5 | 18.7 |
| *P*-value^b^ | 0.8075 |  | 0.2708 |  | 0.2125 |  |
|  |  |  |  |  |  |  |

^a^Percentages have been calculated relative to the number of patients in the population. Summary statistics for the NonDrotAA group have been weighted to reflect the unequal numbers of DrotAA and NonDrotAA patients in each of the matched sets.

^b^Descriptive *P*-values are from clustered binary logistic regression comparing the proportion of patients in the most frequent category between DrotAA vs NonDrotAA, clustering on the matched sets and with weights based on the number of patients in DrotAA and NonDrotAA matched sets. Patients in the unknown categories have been excluded from the tests. No adjustments have been made to account for multiple inference.

1. Use of Vasopressors and Mechanical Ventilation in Matched-INDICATED Primary Analysis Population by IRP B Genotype and Treatment

Percentage of Patients

|  | IRP B+ | | IRP B- | | All Genotypes Combined | |
| --- | --- | --- | --- | --- | --- | --- |
| Supportive Treatment^a^ | DrotAA (n=148) | NonDrotAA (n=424) | DrotAA (n=486) | NonDrotAA (n=1239) | DrotAA (n=639) | NonDrotAA (n=1684) |
|  |  |  |  |  |  |  |
| Use of Vasopressors |  |  |  |  |  |  |
| Yes | 91.9 | 77.6 | 92.4 | 81.8 | 92.3 | 80.9 |
| No | 6.8 | 20.7 | 7.4 | 17.3 | 7.2 | 18.0 |
| Unknown | 1.4 | 1.7 | 0.2 | 0.9 | 0.5 | 1.1 |
| *P*-value^b^ | 0.0004 |  | <.0001 |  | <.0001 |  |
|  |  |  |  |  |  |  |
| Mechanical Ventilation |  |  |  |  |  |  |
| Yes | 68.2 | 78.5 | 77.8 | 75.3 | 75.7 | 76.2 |
| No | 6.8 | 4.2 | 5.6 | 5.3 | 5.8 | 5.1 |
| Unknown | 25.0 | 17.3 | 16.7 | 19.4 | 18.5 | 18.7 |
| *P*-value^b^ | 0.0972 |  | 0.8214 |  | 0.2125 |  |
|  |  |  |  |  |  |  |

^a^Percentages have been calculated relative to the number of patients in the population. Summary statistics for the NonDrotAA group have been weighted to reflect the unequal numbers of DrotAA and NonDrotAA patients in each of the matched sets. ^b^Descriptive *P*-values are from clustered binary logistic regression comparing the proportion of patients in the most frequent category between DrotAA vs NonDrotAA, clustering on the matched sets and with weights based on the number of patients in DrotAA and NonDrotAA matched sets. Patients in the unknown categories have been excluded from the tests. No adjustments have been made to account for multiple inference.

1. Crude Mortality Rates by IRP Genotype and Treatment

Number (%) of Patients

|  | IRP A+ | | IRP A- | | All Genotypes Combined | |
| --- | --- | --- | --- | --- | --- | --- |
| Parameter | DrotAA (n=197) | NonDrotAA (n=517) | DrotAA (n=440) | NonDrotAA (n=1159) | DrotAA (n=639) | NonDrotAA (n=1684) |
|  |  |  |  |  |  |  |
| Deceased | 49(24.9) | 155(30.0) | 111(25.2) | 349(30.1) | 160(25.0) | 505(30.0) |
| ARR^a^ |  | (5.1) |  | (4.9) |  | (4.9) |
|  |  |  |  |  |  |  |

|  | IRP B+ | | IRP B- | |  | |
| --- | --- | --- | --- | --- | --- | --- |
| Parameter | DrotAA (n=148) | NonDrotAA (n=424) | DrotAA (n=486) | NonDrotAA (n=1239) |  |  |
|  |  |  |  |  |  |  |
| Deceased | 36(24.3) | 135(31.8) | 123(25.3) | 359(29.0) |  |  |
| ARR^a^ |  | (7.5) |  | (3.7) |  |  |
|  |  |  |  |  |  |  |

^a^ARR = Absolute Risk Reduction = NonDrotAA mortality – DrotAA mortality.

1. Estimated Weighted Mortality Rates by IRP Genotype and Treatment from Clustered Binomial Regression Model including AIM Panel PCs

Percentage of Patients

|  | IRP A+ | | IRP A- | | All Genotypes Combined | |
| --- | --- | --- | --- | --- | --- | --- |
| Parameter | DrotAA (n=197) | NonDrotAA (n=517) | DrotAA (n=440) | NonDrotAA (n=1159) | DrotAA (n=639) | NonDrotAA (n=1684) |
|  |  |  |  |  |  |  |
| Deceased | 25.3 | 30.3 | 25.1 | 30.8 | 25.1 | 30.5 |
|  |  |  |  |  |  |  |
| ARR^a^ |  | 5.1 |  | 5.7 |  | 5.5 |
| 95% CI |  | (-1.4,11.6) |  | (0.9,10.5) |  | (1.5,9.4) |
| *P*-value^b^ |  | 0.128 |  | 0.020 |  | 0.006 |
|  |  |  |  |  |  |  |

|  | IRP B+ | | IRP B- | |  | |
| --- | --- | --- | --- | --- | --- | --- |
| Parameter | DrotAA (n=148) | NonDrotAA (n=424) | DrotAA (n=486) | NonDrotAA (n=1239) |  |  |
|  |  |  |  |  |  |  |
| Deceased | 24.6 | 32.3 | 25.3 | 29.5 |  |  |
|  |  |  |  |  |  |  |
| ARR^a^ |  | 7.7 |  | 4.2 |  |  |
| 95% CI |  | (-0.7,16.1) |  | (-0.4,8.8) |  |  |
| *P*-value^b^ |  | 0.072 |  | 0.070 |  |  |
|  |  |  |  |  |  |  |

Covariates have been set to their mean values for the Matched INDICATED Primary Analysis Population. Weighted Mortality Estimates reflect the composition of the matched sets; i.e., 1 treated matched with up to 3 control.

A total of 10 AIM Panel PCs have been included which account for 33.9% of the variance in the AIM Panel data for the Matched-INDICATED Primary Analysis Population based on all cohorts.

^a^ARR = Absolute Risk Reduction = NonDrotAA mortality – DrotAA mortality.

^b^*P*-values are from the Wald Chi-square tests for the treatment effect.

1. Secondary Efficacy Analyses for Differential Treatment Effects of IRP A and IRP B on ICU-Free Days through Day 28 – Clustered Poisson Regression Models including AIM Panel PCs

|  | | | | | | |
| --- | --- | --- | --- | --- | --- | --- |
|  | | | | RR 95% CI | |  |
| Factor/Effect | Estimate | SE | RR Estimate | Lower | Upper | *P*-value^b^ |
|  |  |  |  |  |  |  |
| DrotAA vs. NonDrotAA | -0.29 | 0.048 | 0.747 | 0.681 | 0.820 | <0.0001 |
|  |  |  |  |  |  |  |
| IRP A*Treatment Interaction^a^ | 0.03 | 0.107 | 1.033 | 0.838 | 1.274 | 0.7604 |
|  |  |  |  |  |  |  |
| IRP B*Treatment Interaction^a^ | -0.12 | 0.133 | 0.886 | 0.682 | 1.150 | 0.3621 |
|  |  |  |  |  |  |  |

^a^The interaction relative rate (RR) is a ratio of relative rates.

^b^*P*-values are from Wald Chi-square tests.

1. Secondary Efficacy Analyses for Differential Treatment Effects of IRP A and IRP B on Hospital-Free Days through Day 28 – Clustered Poisson Regression Models including AIM Panel PCs

|  | | | | | | |
| --- | --- | --- | --- | --- | --- | --- |
|  | | | | RR 95% CI | |  |
| Factor/Effect | Estimate | SE | RR Estimate | Lower | Upper | *P*-value^b^ |
|  |  |  |  |  |  |  |
| DrotAA vs. NonDrotAA | -0.28 | 0.074 | 0.759 | 0.656 | 0.878 | 0.0002 |
|  |  |  |  |  |  |  |
| IRP A*Treatment Interaction^a^ | 0.06 | 0.167 | 1.061 | 0.764 | 1.472 | 0.7245 |
|  |  |  |  |  |  |  |
| IRP B*Treatment Interaction^a^ | -0.35 | 0.202 | 0.708 | 0.476 | 1.052 | 0.0873 |
|  |  |  |  |  |  |  |

^a^The interaction relative rate (RR) is a ratio of relative rates.

^b^*P*-values are from Wald Chi-square tests.

1. Secondary Efficacy Analyses for Differential Treatment Effects of IRP A and IRP B on Mechanical Ventilator-Free Days through Day 28 – Clustered Poisson Regression Models including AIM Panel PCs

|  | | | | | | |
| --- | --- | --- | --- | --- | --- | --- |
|  | | | | RR 95% CI | |  |
| Factor/Effect | Estimate | SE | RR Estimate | Lower | Upper | *P*-value^b^ |
|  |  |  |  |  |  |  |
| DrotAA vs. NonDrotAA | -0.10 | 0.045 | 0.901 | 0.825 | 0.985 | 0.0222 |
|  |  |  |  |  |  |  |
| IRP A*Treatment Interaction^a^ | -0.01 | 0.102 | 0.991 | 0.811 | 1.212 | 0.9329 |
|  |  |  |  |  |  |  |
| IRP B*Treatment Interaction^a^ | -0.02 | 0.121 | 0.983 | 0.775 | 1.247 | 0.8879 |
|  |  |  |  |  |  |  |

^a^The interaction relative rate (RR) is a ratio of relative rates.

^b^*P*-values are from Wald Chi-square tests.

1. Morbidity Outcomes for Matched-INDICATED Primary Analysis Population by IRP A Genotype and Treatment

|  | IRP A+ | | IRP A- | | All Genotypes Combined | |
| --- | --- | --- | --- | --- | --- | --- |
| Morbidity Parameter^a^ | DrotAA (n=197) | NonDrotAA (n=517) | DrotAA (n=440) | NonDrotAA (n=1159) | DrotAA (n=639) | NonDrotAA (n=1684) |
|  |  |  |  |  |  |  |
| Mechanical-Ventilation-free days through Day 28 |  |  |  |  |  |  |
| N | 156.00 | 387 | 342.00 | 866 | 500.00 | 1261 |
| Mean | 10.95 | 12.42 | 11.24 | 12.43 | 11.15 | 12.42 |
| SD | 10.205 | 6.830 | 10.349 | 6.754 | 10.305 | 6.762 |
| Min | 0.0 | 0.0 | 0.0 | 0.0 | 0.0 | 0.0 |
| Q1 | 0.0 | 0.0 | 0.0 | 0.0 | 0.0 | 0.0 |
| Median | 9.5 | 12.0 | 10.0 | 13.0 | 10.0 | 13.0 |
| Q3 | 21.0 | 23.0 | 21.0 | 23.0 | 21.0 | 23.0 |
| Max | 28.0 | 28.0 | 28.0 | 28.0 | 28.0 | 28.0 |
| *P*-value^b^ | 0.1233 |  | 0.0771 |  | 0.0198 |  |
|  |  |  |  |  |  |  |
| ICU-free days through Day 28 |  |  |  |  |  |  |
| N | 197.00 | 517 | 440.00 | 1159 | 639.00 | 1684 |
| Mean | 7.87 | 10.29 | 7.79 | 10.67 | 7.83 | 10.54 |
| SD | 8.754 | 6.252 | 8.878 | 6.121 | 8.845 | 6.144 |
| Min | 0.0 | 0.0 | 0.0 | 0.0 | 0.0 | 0.0 |
| Q1 | 0.0 | 0.0 | 0.0 | 0.0 | 0.0 | 0.0 |
| Median | 3.0 | 8.0 | 2.0 | 11.0 | 2.0 | 10.0 |
| Q3 | 17.0 | 20.0 | 16.0 | 20.0 | 16.0 | 20.0 |
| Max | 26.0 | 28.0 | 26.0 | 29.0 | 26.0 | 29.0 |
| *P*-value^b^ | 0.0037 |  | <.0001 |  | <.0001 |  |
|  |  |  |  |  |  |  |
| Hospital-free days through Day 28 |  |  |  |  |  |  |
| N | 196.00 | 514 | 437.00 | 1151 | 634.00 | 1671 |
| Mean | 3.93 | 5.10 | 3.61 | 4.93 | 3.74 | 4.96 |
| SD | 6.760 | 4.907 | 6.511 | 4.642 | 6.616 | 4.703 |
| Min | 0.0 | 0.0 | 0.0 | 0.0 | 0.0 | 0.0 |
| Q1 | 0.0 | 0.0 | 0.0 | 0.0 | 0.0 | 0.0 |
| Median | 0.0 | 0.0 | 0.0 | 0.0 | 0.0 | 0.0 |
| Q3 | 7.0 | 10.0 | 5.0 | 9.0 | 5.0 | 10.0 |
| Max | 24.0 | 27.0 | 25.0 | 27.0 | 25.0 | 27.0 |
| *P*-value^b^ | 0.0582 |  | 0.0003 |  | <.0001 |  |
|  |  |  |  |  |  |  |

^a^Percentages have been calculated relative to the number of patients in the population. Summary statistics for the NonDrotAA group have been weighted to reflect the unequal numbers of DrotAA and NonDrotAA patients in each of the matched sets.

^b^Descriptive *P*-values are from clustered Poisson regression comparing DrotAA vs NonDrotAA, clustering on the matched sets and with weights based on the number of patients in DrotAA and NonDrotAA matched sets. No adjustments have been made to account for multiple inference.

1. Morbidity Outcomes for Matched-INDICATED Primary Analysis Population by IRP B Genotype and Treatment

|  | IRP B+ | | IRP B- | | All Genotypes Combined | |
| --- | --- | --- | --- | --- | --- | --- |
| Morbidity Parameter^a^ | DrotAA (n=148) | NonDrotAA (n=424) | DrotAA (n=486) | NonDrotAA (n=1239) | DrotAA (n=639) | NonDrotAA (n=1684) |
|  |  |  |  |  |  |  |
| Mechanical-Ventilation-free days through Day 28 |  |  |  |  |  |  |
| N | 105.00 | 323 | 390.00 | 924 | 500.00 | 1261 |
| Mean | 10.74 | 12.21 | 11.31 | 12.52 | 11.15 | 12.42 |
| SD | 10.069 | 6.891 | 10.398 | 6.743 | 10.305 | 6.762 |
| Min | 0.0 | 0.0 | 0.0 | 0.0 | 0.0 | 0.0 |
| Q1 | 0.0 | 0.0 | 0.0 | 0.0 | 0.0 | 0.0 |
| Median | 8.0 | 12.0 | 11.0 | 14.0 | 10.0 | 13.0 |
| Q3 | 20.0 | 24.0 | 21.0 | 23.0 | 21.0 | 23.0 |
| Max | 28.0 | 28.0 | 28.0 | 28.0 | 28.0 | 28.0 |
| *P*-value^b^ | 0.2303 |  | 0.0580 |  | 0.0198 |  |
|  |  |  |  |  |  |  |
| ICU-free days through Day 28 |  |  |  |  |  |  |
| N | 148.00 | 424 | 486.00 | 1239 | 639.00 | 1684 |
| Mean | 6.95 | 10.32 | 8.13 | 10.69 | 7.83 | 10.54 |
| SD | 8.635 | 6.366 | 8.923 | 6.093 | 8.845 | 6.144 |
| Min | 0.0 | 0.0 | 0.0 | 0.0 | 0.0 | 0.0 |
| Q1 | 0.0 | 0.0 | 0.0 | 0.0 | 0.0 | 0.0 |
| Median | 0.5 | 8.5 | 3.0 | 11.0 | 2.0 | 10.0 |
| Q3 | 14.5 | 21.0 | 17.0 | 20.0 | 16.0 | 20.0 |
| Max | 25.0 | 27.0 | 26.0 | 29.0 | 26.0 | 29.0 |
| *P*-value^b^ | 0.0009 |  | <.0001 |  | <.0001 |  |
|  |  |  |  |  |  |  |
| Hospital-free days through Day 28 |  |  |  |  |  |  |
| N | 146.00 | 421 | 483.00 | 1229 | 634.00 | 1671 |
| Mean | 2.86 | 5.07 | 4.04 | 4.95 | 3.74 | 4.96 |
| SD | 5.647 | 4.936 | 6.886 | 4.649 | 6.616 | 4.703 |
| Min | 0.0 | 0.0 | 0.0 | 0.0 | 0.0 | 0.0 |
| Q1 | 0.0 | 0.0 | 0.0 | 0.0 | 0.0 | 0.0 |
| Median | 0.0 | 0.0 | 0.0 | 0.0 | 0.0 | 0.0 |
| Q3 | 1.0 | 10.0 | 7.0 | 9.0 | 5.0 | 10.0 |
| Max | 23.0 | 27.0 | 25.0 | 27.0 | 25.0 | 27.0 |
| *P*-value^b^ | 0.0030 |  | 0.0153 |  | <.0001 |  |
|  |  |  |  |  |  |  |

^a^Percentages have been calculated relative to the number of patients in the population. Summary statistics for the NonDrotAA group have been weighted to reflect the unequal numbers of DrotAA and NonDrotAA patients in each of the matched sets.

^b^Descriptive *P*-values are from clustered Poisson regression comparing DrotAA vs NonDrotAA, clustering on the matched sets and with weights based on the number of patients in DrotAA and NonDrotAA matched sets. No adjustments have been made to account for multiple inference.

**SUPPLEMENT REFERENCES**

1. Annane D, Mira JP, Ware LB, et al. Design, conduct, and analysis of a multicenter, pharmacogenomic, biomarker study in matched patients with severe sepsis treated with or without drotrecogin Alfa (activated). *Annals of intensive care.* 2012;2(1):15.
2. Knaus WA, Draper EA, Wagner DP, Zimmerman JE. APACHE II: a severity of disease classification system. *Crit Care Med.* Oct 1985;13(10):818-829.
3. Le Gall JR, Lemeshow S, Saulnier F. A new Simplified Acute Physiology Score (SAPS II) based on a European/North American multicenter study. *Jama.* Dec 22-29 1993;270(24):2957-2963.
4. Austin PC. A critical appraisal of propensity-score matching in the medical literature between 1996 and 2003. *Statistics in medicine.* May 30 2008;27(12):2037-2049.
5. Kosoy R, Nassir R, Tian C, et al. Ancestry informative marker sets for determining continental origin and admixture proportions in common populations in America. *Hum Mutat.* Jan 2009;30(1):69-78.
6. Nassir R, Kosoy R, Tian C, et al. An ancestry informative marker set for determining continental origin: validation and extension using human genome diversity panels. *BMC Genet.* 2009;10:39.
